# Machine learning model for sequence-driven DNA G-quadruplex formation

**DOI:** 10.1038/s41598-017-14017-4

**Published:** 2017-11-06

**Authors:** Aleksandr B. Sahakyan, Vicki S. Chambers, Giovanni Marsico, Tobias Santner, Marco Di Antonio, Shankar Balasubramanian

**Affiliations:** 1 Department of Chemistry, University of Cambridge, Lensfield Road, Cambridge, CB2 1EW UK; 2 Cancer Research UK Cambridge Institute, University of Cambridge, Li Ka Shing Centre, Robinson Way, Cambridge, CB2 0RE UK; 3 School of Clinical Medicine, University of Cambridge, Cambridge, CB2 0SP UK

## Abstract

We describe a sequence-based computational model to predict DNA G-quadruplex (G4) formation. The model was developed using large-scale machine learning from an extensive experimental G4-formation dataset, recently obtained for the human genome via G4-seq methodology. Our model differentiates many widely accepted putative quadruplex sequences that do not actually form stable genomic G4 structures, correctly assessing the G4 folding potential of over 700,000 such sequences in the human genome. Moreover, our approach reveals the relative importance of sequence-based features coming from both within the G4 motifs and their flanking regions. The developed model can be applied to any DNA sequence or genome to characterise sequence-driven intramolecular G4 formation propensities.

## Introduction

**Figure 1:**
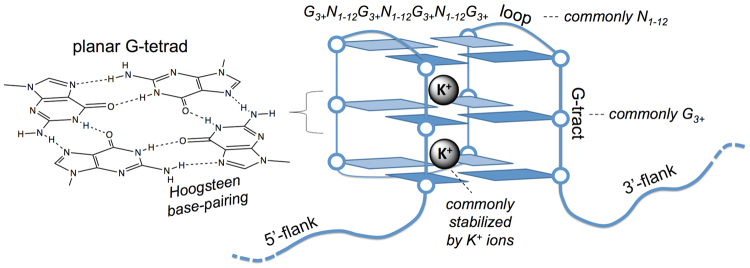
Schematic representation of features of canonical G-quadruplex (G4) structures in DNA, along with a common sequence motif used to identify such structures. The structure comprises four tracts of guanines (G-tracts) that form planar G-tetrads through Hoogsteen base-pairing.

G-quadruplex structures (G4s) are alternative DNA conformations with an increasing body of evidence for their functional role and influence in living cells^1–5^. G4s are typically formed by guanine tracts interspersed with three loops and stabilised through stacked, Hoogsteen base-paired, planar G-tetrads (Fig. 1).

Despite significant experimental advances in the exploration of G4s^2,3,6–8^, the computational framework for G4 prediction has remained mostly at the level of simple bioinformatics motif analysis^9–11^ (G_3+_N_1−n_G_3+_N_1−n_G_3+_N_1−n_G_3+_, referred to as putative quadruplex sequence - PQS, see Methods). While attempts have been made to address stability scoring in such motifs, the current models rely on considerations of simple characteristics (lengths of the G-tracts, the loop sequences, G-skewness) or biophysical measurements for short sequences that lack their wider genomic context^1,12–16^. Furthermore, the absence of large biophysical datasets for G4-forming sequences, has hitherto precluded a more complete sequence-based model for G4 stability.

G4-seq, an experimental approach to identify G4s in a genome-wide manner in human genomic DNA, has recently been published^8^. The method exploited G4-specific polymerase stalling to detect G4s in single-stranded human genomic DNA. When carried out directly on the Illumina sequencing flow cell, the method allowed the high-throughput assessment of millions of sequences simultaneously. The output of G4-seq is a profile of base mismatch levels (*mm%*) for the whole genome, whereby a higher *mm%* is indicative of a more stable G4^8^.

In this work, given the scale of the available G4-seq dataset and the recent success of large-scale machine learning approaches in deciphering complex genomic dependencies^17–19^, we sought to develop a machine learning procedure to build a G4-formation model based on a multitude of sequence-only features (see Methods, Supporting Information Figures S1–S9). The employed approach allowed a joint consideration and optimisation of features, without any analytical pre-assumption on the way the features should interact with each other to produce the outcome predictive model. Moreover, part of the used features stemmed from within flanking regions around potential G4 sequences, which were previously highlighted as important contributors to G4 formation and stability^20^. For the sequence-based G4 prediction problem, it is relatively straightforward to devise models that would have either high sensitivity (hence detecting most G4 forming sequences, in addition to many potential false positives), or high specificity (hence excluding most sequences that cannot form G4s, in addition to many potential false negatives). The major challenge here is achieving a combination of high sensitivity with high specificity, which we solve here for the clearly defined and major part of the universe of G4 forming sequences.

## Results and Discussion

### Source data and general approach for model development

**Figure 2:**
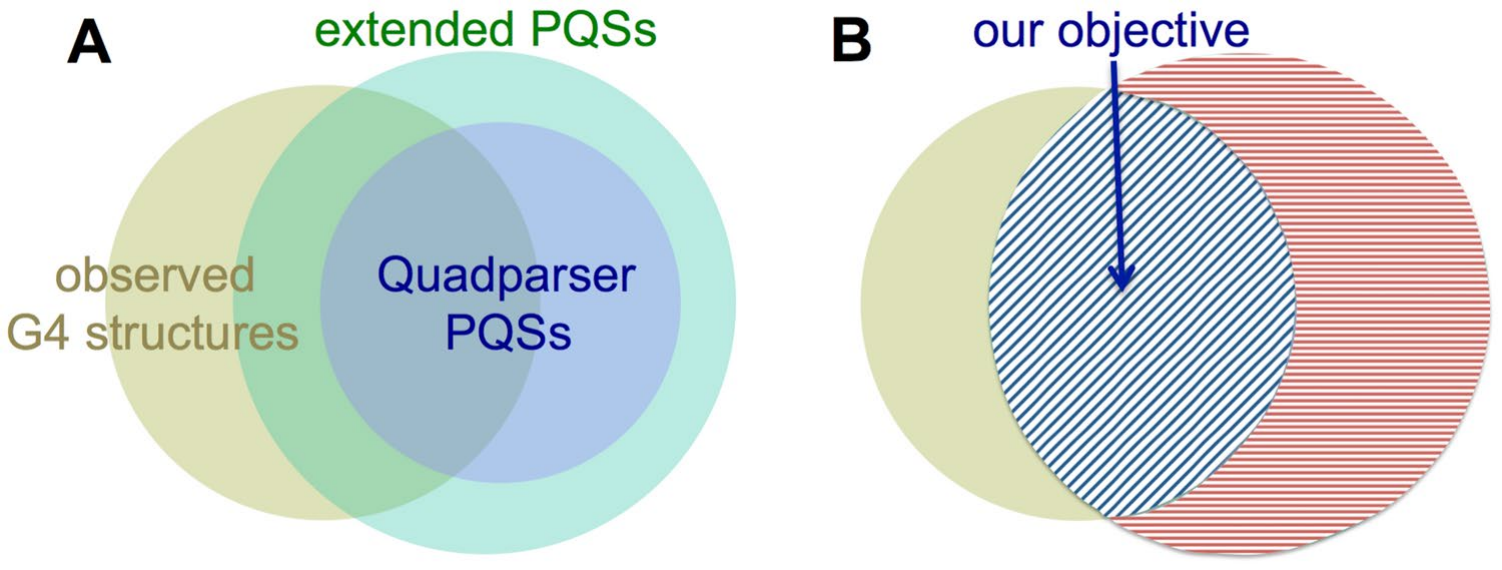
Euler diagrams showing the overlap between the experimentally observed G4 structures (dark yellow disc) and the putative quadruplex sequences (PQSs) found via simple sequence motif search in the human genome. The violet disc in (**A**) represents the more conservative Quadparser (G_3+_N_1−7_G_3+_N_1−7_G_3+_N_1−7_G_3+_) sequences. The green disc in (**A**) represents the extended sequence motif with longer allowed maximum loop size - G_3+_N_1−12_G_3+_N_1−12_G_3+_N_1−12_G_3+_. Both motifs result in similarly high (46.37% and 50.96%) false positive rates, however, the extended motif covers a bigger portion of experimentally observed G4 structures (65.56% vs. 36.86%). (**B**) Represents the objective of our present work, which is to develop a machine learning model that, starting from the extended PQS motif definition, would correctly differentiate sequences that form stable G4 structures (blue-shaded overlap in (**B**)) from the ones that do not (red shaded part in (**B**)).

We started from the available experimental G4-seq *mm%* profile for the human genome (see Methods). The overlap between G4-seq experimentally observed G4 structures and putative quadruplex sequences (PQSs), that are based on bioinformatics motif search in the human genome (Fig. 2A), indicate that simple computational methods result in many sequences that do not actually form stable G4s (parts of green and violet discs in Fig. 2A not overlapped with the dark yellow one), despite possessing the canonical set of four G-tracts (Fig. 1).

On the other hand, the observed G4s that do not overlap with PQSs (parts of the dark yellow discs in Fig. 2) are comprised of non-canonical G4 sequences. Those sequences have bulges and/or G-tracts with only two guanines, hence differ from the standard, canonical PQS motif presented in Fig. 1. We considered both the more stringent, Quadparser^9^, definition of PQSs (G_3+_N_1–7_G_3+_N_1–7_G_3+_N_1–7_G_3+_) and an extended version of PQSs allowing longer loops (G_3+_N_1–12_G_3+_N_1–12_G_3+_N_1–12_G_3+_), to see whether the choice of the PQS definition would affect our observations. However, we still noticed a large fraction of PQSs (Fig. 2) not detected as stable G4s by either class of sequence motifs (46.37% and 50.96%, for stringent Quadparser and extended PQSs respectively), while the extended PQS definition covered a greater fraction of experimentally observed G4s (65.56% for extended vs. 36.86% for stringent PQS definitions). The extended PQSs have thus been used preferentially for our further studies.

We have initially attempted to develop a *de novo* model that does not depend on any initial PQS definition. Owing to the resolution of the G4-seq technique, which is comparable to the length of G4 sequences, for non-canonical G4 structures it is difficult to define the sequence boundaries and differentiate G4 features from the flank features as sources to base the predictions on (see Methods for details). We therefore started from the extended PQS definition that includes the 65.56% canonical subset of observed G4s (the overlap area between the dark yellow, observed G4, and green, extended PQS, disks in Fig. 2A, blue shaded in Fig. 2B), and developed a machine learning model that would correctly identify the stable G4s with improved false positive (FPR) and false discovery (FDR) rates (see Methods) compared to available G4 prediction methods.

**Figure 3:**
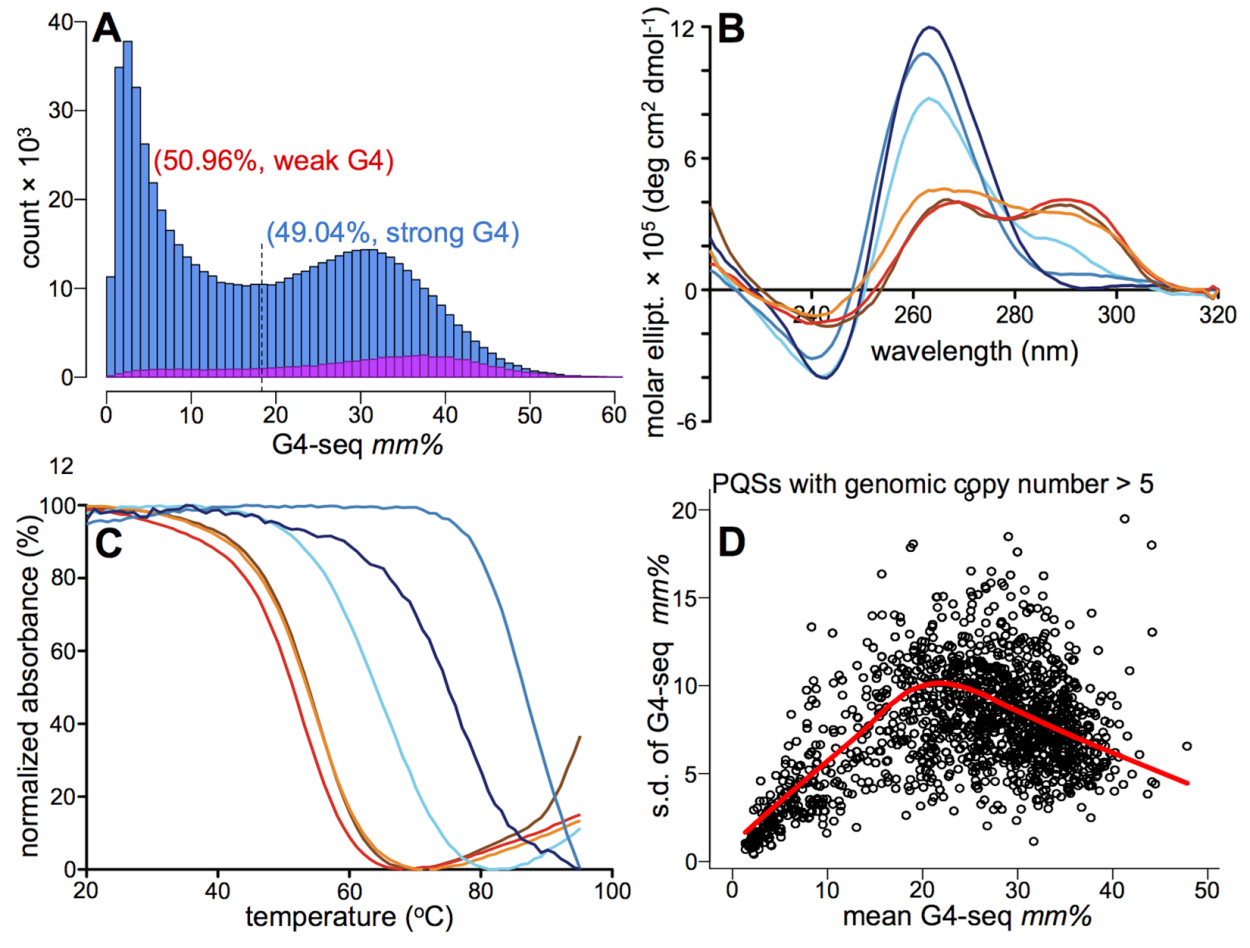
Canonical putative quadruplex sequences do not necessarily form stable genomic G4s. (**A**) The distribution of *mm%* values for PQSs, highlighting the presence of two clusters. (**B**) Example CD spectra for sequences from the weak- (shades of red) and strong-G4 (shades of blue) clusters (as judged from G4-seq *mm%* values). The CD signature for G4 comprises ~260 nm maximum and ~240 nm minimum for parallel topology, or ~290 nm maximum and ~260 nm minimum for antiparallel topology^42,43^. (**C**) The example UV-melting (for 295 nm) spectra from the same sequences in (**B**). The chosen sequences with their respective *mm%* and T_m_ (UV melting temperature) values are highlighted in Table S1, using the same colour code. The increased absorbance for some samples, after the full melting of G4 structures, is most probably due to a partial evaporation from the solution during the continuous measurement at high temperatures. (**D**) The G4-seq *mm%* variance vs. mean value dependence for PQSs with multiple genomic copy numbers, where only the flanking sequences are different, to directly demonstrate the role of flanks on G4 formation propensity. See also Figures S10–S14, Tables S1 and S2.

Based on the experimental G4-seq *mm%* values (Figure S10) for the 703,091 human canonical PQSs (see Methods)^6^, we found that 50.96% of such widely-used sequence motifs actually did not form stable genomic G4 structures (have low *mm%*)^8^ (Fig. 3A, see Methods). Moreover, data shows two distinct clusters for PQSs (Fig. 3A) defining non-existent/weak and strong G4s. None of the simple sequence characteristics were sufficient to explain such stability differences between the two clusters (see Methods), suggesting the need for machine learning to capture the possible non-additive and highly multiplexed dependence between the sequence-based features and G4 stabilities. We therefore developed such a data-driven procedure for predicting G4s with the aim of providing improved agreement with experimental outcomes with regard to G4 stability.

### The employed machine learning strategy

We selected the tree-based gradient boosting machines (GBMs)^21–23^ to be our central framework for the model development (see Methods). Gradient boosting is a powerful technique, where an ensemble of learners is generated, with each consecutive learner trying to predict the residual of the ensemble of prior learners^21–24^. The methodology is rather flexible, providing a number of parameters to tune in order to get the optimal architecture tailored to a particular problem^22,24^. Although, in principle, gradient boosting can be applied to any learning methodology, decision trees are the base learners most frequently used with gradient boosting, due to the leading performance of the combination^22–25^. The underlying decision trees (number of trees, interaction depth, minimum child weight) and the gradient boosting procedure itself (shrinkage or learning rate, resampling or subsampling ratio, also known as bag fraction) use a number of tuneable parameters (5 used herein, as listed in the brackets above) to define the overall architecture of the method, which can be specifically adapted to its maximum performance for a given problem. Tree-based GBMs are considered to be amongst the top-performing machine learning methodologies providing the best results, either alone or in mixtures with deep learning, in a wide range of competitions (http://www.kaggle.com/) and predictive modelling objectives^23,26,27^. Furthermore, the tree-based nature of the learners enables the extraction of indirect information on the importance of different features for the overall prediction quality (as discussed below), thus providing semi-transparent means of having structural insight into the complex model, not easily available in other high-performing machine learning approaches.

We exploited the human genome G4-seq dataset, which provides an extensive set of PQSs localised within sequence contexts with varying degrees of G + C content. This ensured the presence of a compositionally diverse training set for a transferrable model. We first carried out exploratory data analyses to select the optimal machine learning strategy for the model development. We confirmed that the *mm%* values indeed reflected the stability variation of the G4-sequences assessed biophysically by circular dichroism (CD),^1^H NMR and UV-melting spectroscopic techniques (see Methods, Figures S11–S14, Tables S1 and S2). Sequences selected from the weak-G4 (i.e. less stable) cluster showed weak biophysical G4 signatures, contrasting to the sequences selected from the strong-G4 cluster (examples in Fig. 3B,C). These observations demonstrate the hierarchical link between the *mm%* values and the structural stability of G4s (Figure S13) and led us to choose a regression type machine learning procedure, to predict the experimental *mm%* values, as opposed to a simpler classification that would just predict whether a given sequence is a stable G4 or not.

### Sequence-based features

A supervised machine learning procedure requires training data, where each entry contains both the necessary response value (*mm%*), and the set of features (in our case, derived from only a DNA sequence) to build the model upon. Taking into account evidence that flanking sequences affect the stability of G4s^20^, we first examined the role of the flanking regions in defining the structural state of PQSs. By using those PQSs that were present in the human genome in multiple copies (same G4 sequence but with varying flanks), we investigated the dependence of the standard deviation of G4-seq *mm%* values for such sequences (with at least 5 copies across the human genome) from the mean *mm%*. The outcome directly shows (Fig. 3D, Figure S14, Table S2) that the flank-dependent standard deviations may result in *mm%* variation of half the magnitude of the mean *mm%* for the low-stability G4s (see the red Lowess trendline in Fig. 3D). The behaviour is however inverted starting from ~20 *mm%* value, with the standard deviation gradually decreasing to up to 1/10 of the mean *mm%* for the stable G4s (Fig. 3D). While demonstrating the overall influence of the flanks in modulating G4 stability^20^, simple motif analyses aimed at finding associated flank features resulted in no conclusive outcome (Figure S15). This prompted our decision to apply advanced high-end machine learning methodologies, while also considering sequence-based features from G4 flanks. 209 sequence-based features, stemming from both PQSs and flanks, were thus selected for consideration in our model development, as detailed in the Methods section and Figure S1b,c. 201 were the triad and singleton contents of PQS and its 5′- and 3′-flanks, considered separately. 8 other features reflected the overall length and loop characteristics of the PQS moiety, without the flanks.

### Optimisation of the machine learning architecture and intrinsic performance metric

**Figure 4:**
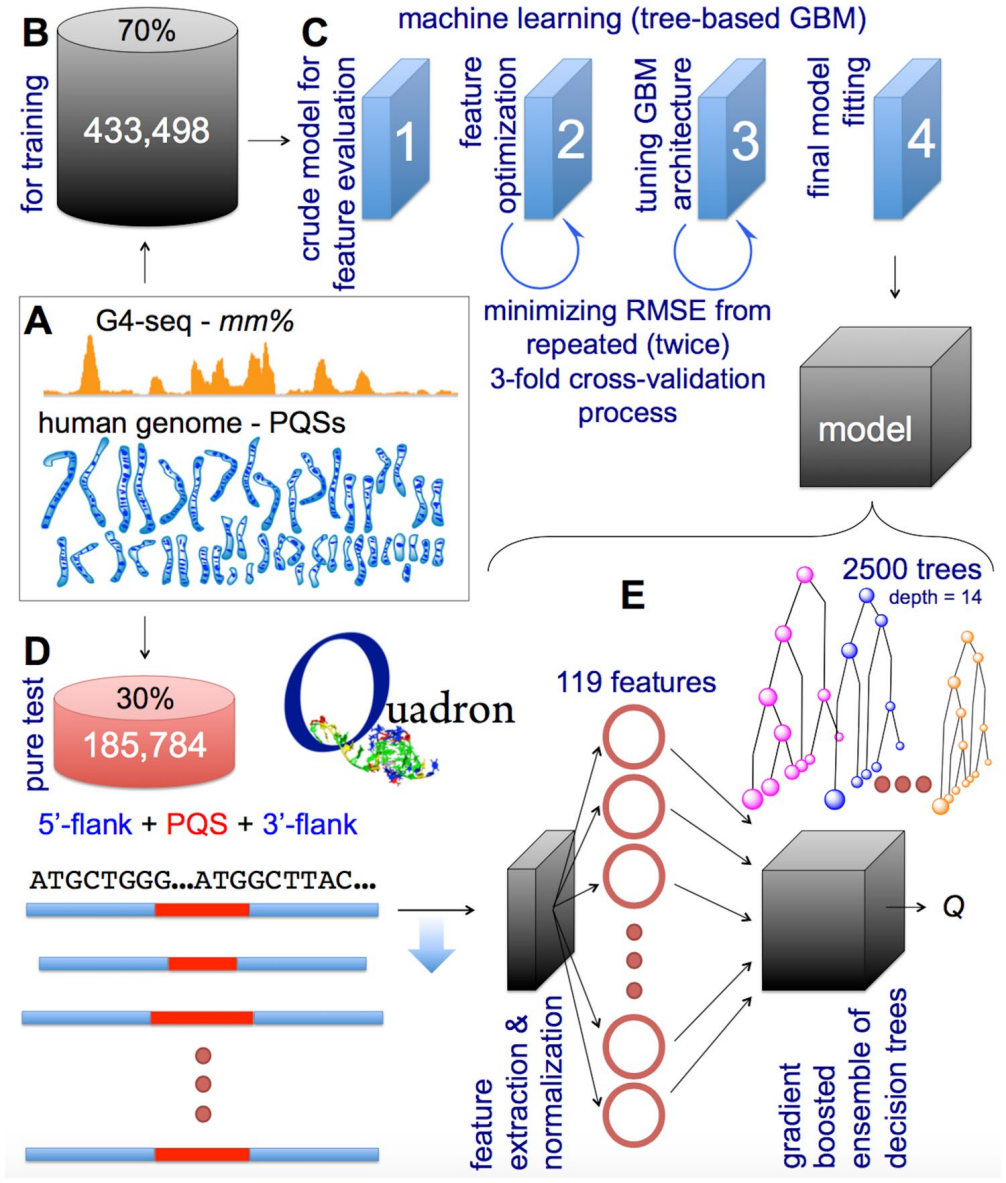
The developed machine learning workflow behind the Quadron model generation. The source data (**A**), its partition used for training cycles (**B**), the major stages of the machine learning procedure (**C**), and the partition of the source data used for model testing (**D**) are schematically shown, along with the structure of the outcome model (**E**), further detailed and referenced in the main text.

For tuning the machine learning architecture, we used extended PQSs found throughout the human genome, along with their flank sequences and experimental *mm%* values associated with each PQS (Fig. 4A). We partitioned the data and used only 70% to tune the GBM architecture and train the model (Fig. 4B, Figure S2), leaving the remaining 30% to serve as an external validation set for the final model. Our source data represented G4s of varying stability, with nearly equal numbers of sequences in the weak-G4 and strong-G4 clusters (Fig. 2A). We employed a workflow for the subsequent feature set optimisation and GBM architecture tuning (Fig. 4C, Figures S3-S9) driven by a performance metric (root mean squared error) coming from a 3-fold cross validation repeated 2 times (see Methods, Figure S4). At a given instance, 2/3 of the training data was used for actual training while 1/3 was used for the internal testing (not to be mistaken with the 30% external test dataset). Such large-chunked divisions within internal cross-validation cycles ensured minimal PQS redundancy between internal testing and training partitions, as only ~7% of PQSs in the internal test partition were repeated in the training one, however, still with varying flank sequences.

### The final model, external validation and characteristics

The final model (Fig. 4E), named Quadron, which arose from the application of the optimal GBM architecture on the whole training data (see Methods), consists of a module that identifies all the PQS motifs in the provided sequence, computationally “digests” each PQS along with the 50-nt long 5′- and 3′-flanks to result in 119 optimal sequence-based features (from the initially benchmarked 209). It then passes the values of all the features onto the gradient boosted ensemble of 2500 decision trees, resulting in stability scores predicted solely based on the sequence.

**Figure 5:**
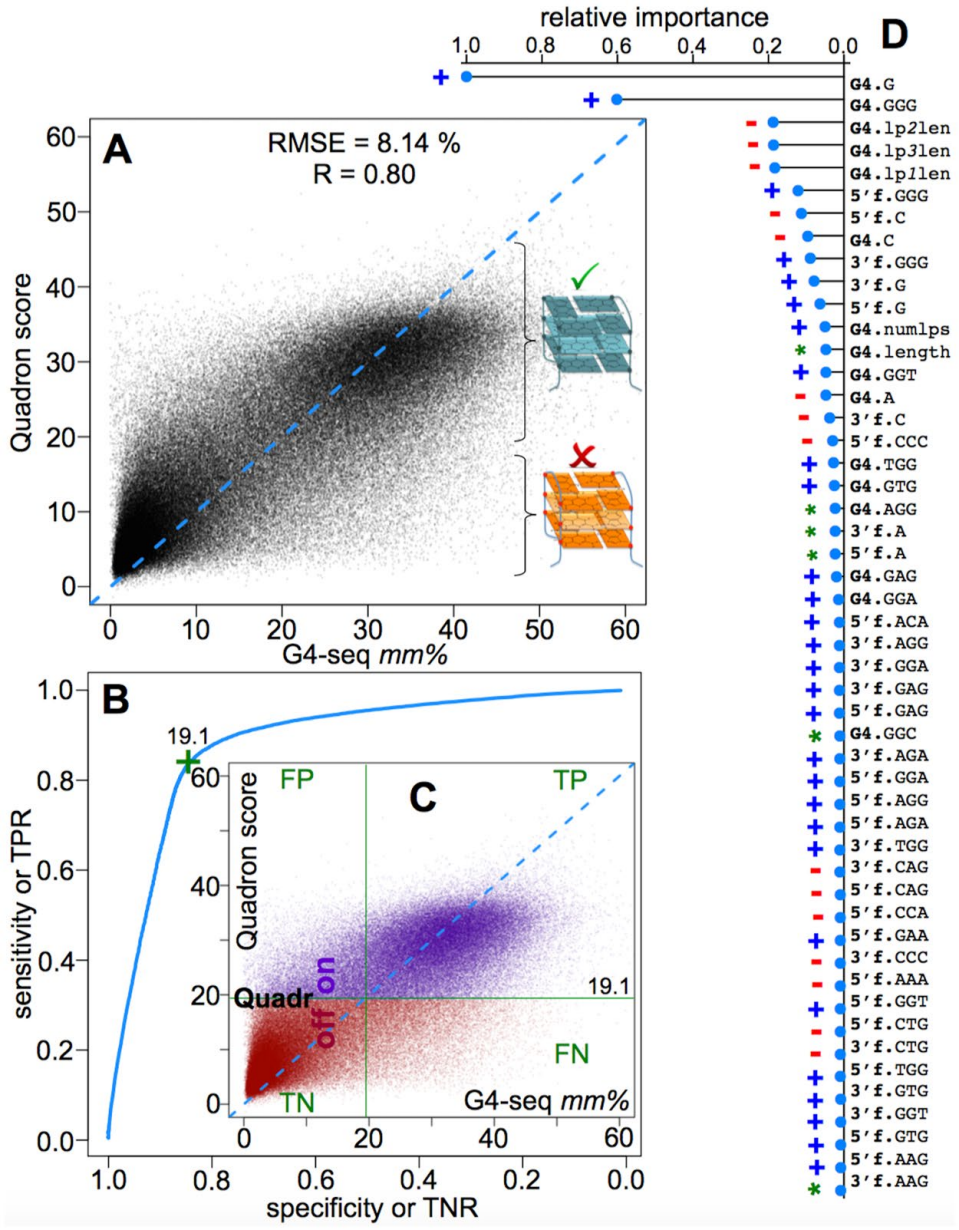
The performance of the final model. (**A**–**C**) show the Quadron performance against the experimental G4-seq dataset not used in the model development (correlation - **A**; classification – (**B**) and (**C**). (**D**) presents the top 50 sequence-based features ranked by relative importance (fully listed in Table S3) for the achieved prediction quality. The feature names are explained in Methods. Blue (+), red (−) and green (*) marks highlight the features as generally G4-stabilising, generally G4-destabilising, and more complex respectively (Methods).

Quadron was then applied to the external validation dataset (30% of the original G4-seq data left out from the beginning, Fig. 4D) and the outcome is shown in Fig. 5A. A strong agreement between prediction and measurement can be noted with 0.80 Pearson’s correlation coefficient and 8.14 root mean squared error (in G4-seq *mm%* unit). Furthermore, the strong- and weak-G4 clusters are well differentiated by Quadron, where we can reach 84.2% true positive rate (TPR), 84.1% true negative rate (TNR), 15.9% false positive rate (FPR) and 17.5% false discovery rate (FDR), while using 19.1 as the *mm%* threshold (Fig. 5B,C, see Methods).

Next, we explored the role of different sequence-based features in defining the success of our model (see Methods, Table S3). This was done via the relative feature importance values that the tree-based GBM provides as an indirect metric to reflect upon the number of times a given feature was used for a split in the underlying decision trees, along with the squared improvement achieved in describing (fitting) the training data after such splits^22,24^. The top 50 most influential features are summarised in Fig. 5D, and contain features derived from both the G4 sequence and its flanks (Figure S16). We can see that extra G-tracts in the flanks are rather influential, along with the GGW, GWG and WGG (W = {A,T}) triads in both G4s and their flanks. Overall, G, GGG, C and CCC contents in G4s and flanks together account for 59.4% of the performance of Quadron (as judged from the share in the summed importance scores brought in Table S3), while the remaining 40.6% improvement in the model is due to interdependencies in much weaker features, which we were able to capture owing to the used machine learning framework.

### Application of Quadron on another genome

We have used the genome of *C. elegans* (assembly WS220 taken from http://www.wormbase.org), as a different genome where no G4-seq experimental data is available, hence Quadron can be useful *per se* for applicability demonstration. From all Quadron extracted sequences and associated scores, we randomly selected examples with varying Quadron scores (Table S4). The actual sequences (without flanks) were then ordered and biophysically studied through the UV-melting experiments (see Methods). Even though the biophysically-tested short, G4-only, sequences are devoid of influential flanking sequences and are thus a rather coarse reflection of G4 states in their natural genomic context, on average, the results still show an agreement between the Quadron prediction of the hierarchy of G4 stability (accounting for genomic context) and the data from biophysical characterisation (Figure S17).

### The stand-alone program implementation

The stand-alone implementation of the obtained model has been publicly released as the program Quadron (http://quadron.atgcdynamics.org), using *R* and its *xgboost* library, which makes the eXtreem Gradient Boosting^23^ (http://github.com/dmlc/xgboost) available in *R*. An additional browser-based graphical user interface is written with Shiny (http://shiny.rstudio.com), also enabling an on-line server usage of the program (Figure S18). As an output, the program returns the list of detected PQSs from both strands of the provided DNA sequence, along with their length, coordinates, and the predicted Quadron score, defining whether a given PQS would or would not form a stable genomic G4 structure.

## Conclusion

In summary, Quadron enables the extraction of information about G4 formation and stability from the DNA sequence only, by exploiting the complex links learned from the massive genome-wide G4-seq dataset. Quadron correctly predicts the G4 formation propensities, with Pearson’s R = 0.8 and RMSD = 8.14 *mm%* for the test set in the human genome. Crucially, our model reflects the bimodal nature of putative quadruplex sequences (PQSs) and accurately discriminates (TPR = 84.2%, TNR = 84.1%, FPR = 15.9%, FDR = 17.5%) between PQSs that can or cannot form stable G4. It therefore reveals the significant proportion of PQSs (50.96% in the human genome) with weak intrinsic potential for G4 formation - a subset often neglected by conventional simpler predictors.

Quadron highlights the complex nature of how an ensemble of simple sequence-based features (weak predictors, Fig. 5D), many without any direct correlation with G4-formation propensity (Figure S16, Table S3), can produce a powerful model while their fine interactions are captured *via* an advanced machine learning approach. In fact, accounting for only G- and C-content based features (GGG and CCC triplets inclusive) would result in 59.4% of Quadron’s performance at best, even while their multidimensional interconnection is exploited through the ensemble of gradient boosted trees. The rest of the features, with their complex inter-dependencies, are therefore essential in the success of our model.

Whilst a number of extra-sequence features, such as the consequences of chromatin folding and proteins^4^ or DNA methylation^28^, can influence G4 formation in a cellular context, Quadron focuses on only the sequence-driven intrinsic capability of DNA to form G4s. Extra-sequence features are subject to variation depending on the studied species, cell type and cell cycle stage, while the intrinsic propensity of DNA to form G4s contributes to the *in vivo* G4 formation to the same extent, independently of the cellular identity and state. Our model therefore provides important and transferrable information to explore G4 formation potential in any DNA sequence or genome.

We expect Quadron could be used for predicting extended canonical G4 structure formation propensities in any DNA sequence or genome where demanding and expensive experimental G4-seq data are absent. The work is underway to extend our predictions towards the non-canonical G4 sequences, while still keeping the stringent standards on the usability metrics (TPR, FPR, FDR) of the model.

### Data Access

Quadron is freely accessible (Methods, Figures S9 and S18 **)** from http://quadron.atgcdynamics.org. Quadron is available both as a server application and as a stand-alone program. The stand-alone version can be executed through the command line, useful while incorporating Quadron in automatic workflows, or through its graphical user interface. The source code is also freely available through GitHub (http://github.com/aleksahak/Quadron), distributed under the version 2 of the general public license (GPL v.2). Both the program and the source code can be accessed *via* http://quadron.atgcdynamics.org.

## Methods

Below is the description of the computational and experimental procedures, cross-referenced to further details in Supporting Figures, Tables and their captions. The numbering and order of the Supporting Figures and Tables, additionally cited in Methods below, is in correspondence with the main text of the paper, hence their referencing in this section may not appear sequentially.

### General notes on the performed computations

The workflows used in this study were built *via* the *R* programming language^29^. The large-scale machine learning^17,18,30,31^ optimisations were done using the computing cluster at the Cancer Research UK Cambridge Institute, employing the nodes with 96.7 GB of random access memory and 2 × Intel Xeon X5650 processors (totalling to 12 computing cores) per node. The analyses involving the human genome were based on the reference sequence of version hg19/GRCh37, as retrieved from the Ensembl genome database (http://www.ensembl.org).

### Experimental G4-seq data for the human genome

For the exploratory data analyses, machine-learning procedure, and testing of the resulting model, we used the only available G4-seq data for the human nuclear genome^8^. The G4-seq experiment identifies G-quadruplex structures (G4s) in a genome-wide high-throughput manner^8^. The method exploits G4-specific polymerase stalling and outputs a profile of base mismatch levels (*mm%*) for the whole genome, with a higher *mm%* indicative of more stable G4s^8^. The experimental G4-seq data, in the form of the average mismatch levels (*mm%*) per 15-nt-long DNA sequence (with sequencing coverage) had been generated for the entire human genome, and were accessed through the GEO repository (accession number: GSE63874). We took the data for the Na^+^ vs. K^+^ gradient (as opposed to the Na^+^ vs. pyridostatin (PDS) gradient) experiments^8^, since the chosen ions that contrast the G4-formation conditions are those present in living cells. Furthermore, the nature of G4-seq experiment, where the values are corrected against the background control experiment, eliminates possible biases in polymerase stalling that might be present because of G + C content variation, or any other artefacts dependent on a sequence, rather than G4 structure.

The human genome is a reasonable experimental source for developing a transferrable computational model for G4 formation, as it presents many G4 structures and G4-like sequences to train and test data on. Furthermore, the G + C content of megabase-long stretches within the human genome is markedly variable^32^, presenting pools of G4 motifs characteristic of varying degrees of base content.

### *De novo* general approach for machine learning with G4-seq

Having the complete G4-seq profile for the human genome, we were first tempted to develop a general machine learning model to target the link between any DNA sequence and G4-seq measured *mm%*. Such a model was appealing as would include non-canonical quadruplex sequences that are out of the scope of any frozen initial motif definition. However, the approach did not result in a model with significant predictive power in validation tests. This was because of the previously noted characteristic of G4-seq technique^8^, whereby it produces peaks of base mismatch levels (*mm%*) at the vicinity, rather than exactly on, G4-forming sequences. Since our observed *mm%* values can be characteristic to any G4 structure within 50-nt range, we had to take 50–100-nt long DNA sequence bins to surely engulf a potential G4 that may give rise to an observed *mm%* value (from the G4-seq experiments) for each such sequence span. The required bins were thus much longer than the average length (~34 nt) of actual G4 forming sequences. To this end, when we derived sequence-based features from such bins, even the ones that held real G4 structures still contained 2–3 times longer non-G4 DNA. The G4-positive bins therefore produced sequence-based features not much different from the features of the bins devoid of G4s. This was the major factor that prevented our machine learning procedure to produce an acceptable predictor of *mm%* values in this general approach.

### The chosen specific approach for machine learning with G4-seq

Because of the considerations from the generalised strategy detailed above, we instead focused on the task of quantifying the G4-formation propensities for the sequences from the putative quadruplex sequence (PQS) “universe”, where specific sequence motifs, hence borders, can be assigned to potential G4 structures. Despite the widely accepted use of the PQS definition^9–11^ (see the next subsection) and the expectation that most of such canonical sequence motifs form stable G4 structures, PQSs still carry the surprising issue of containing many sequences (50.96% in the human genome, Fig. 3A,B) that do not actually form stable genomic G4 structures as found through G4-seq experiments and our further validation (Fig. 3A–D, Figures S10–S13, Table S1)^8^. Therefore, being able to increase the precision of identifying stable G4 structures from within the PQS “universe” is still a major undertaking (see the main text), which has resulted in a positive outcome as detailed in our work.

### Putative quadruplex sequence (PQS) motifs

Putative quadruplex sequences (PQSs) were defined through the G_g1_N_x_G_g2_N_y_G_g3_N_z_G_g4_ general motif^9^, where g1, g2, g3 and g4 are integer numbers equal to or greater than 3; x, y and z are integers between 1 and L^max^ (inclusive); N is any nitrogenous base, including guanine. For the retrieval of the *in silico* putative quadruplex sequences, we used the *extended* definition with the maximum loop size of 12 (L^max^ = 12), increasingly used^11^ after growing evidence for the existence of G4s with loops longer^33–35^ than the traditionally quoted 7 nucleotides^9,10^. The sequences were retrieved *via* a regular expression search within each human nuclear chromosome. For the nested PQSs, the longest sequence engulfing the constituent PQSs was retrieved. Therefore, the motif searched within genomes was {G_3+_N_1−12_}_3+_G_3+_, which would identify longest possible DNA stretches with G-tracts lengthier than 2 nucleotides each, interspersed with loops shorter than 13 nucleotides in length that may contain Gs, but not G_3+_ tracts within. This resulted in the identification of 703,091 PQSs, similar in number to that reported elsewhere^11^.

### Mapping of the G4-seq mismatch levels to the human PQS dataset

The G4-seq data were mapped to the positions of the 703,091 *in silico* PQSs spans in the human genome. For the robust mapping, we used the 5′- and 3′-end coordinates for each identified PQS, allowing additional 50-nt-long 5′- and 3′-flanks around PQSs (Figure S1a,b). The consideration of the regions covering the flanks was done to account for the fact that the polymerase-stalling-induced base mismatches in G4-seq normally happen near the actual G4 structures^8^, and the maximum mismatch levels (*mm%*) are normally observed somewhere within 50-nt range from the G4 (see subsection ‘De novo general approach for machine learning with G4-seq’). After the PQS and G4-seq data cross-mapping, for each PQS we took the maximum G4-seq *mm%* from the associated 15-nt-long DNA segments that overlap with the PQS and flank regions (Figure S1a,b). Therefore, this procedure provided a single unique *mm%* value per PQS site, which could then be used as an observation for the consecutive machine learning purposes. 687,846 out of 703,091 PQSs gained unique *mm%* values, with 15,245 left out (Figure S2) due to the lack of G4-seq coverage at their sites in the human genome.

### Exploratory data analysis on the experimental G4-seq data

Prior to the machine learning procedure, we had performed exploratory data analysis on the eligible 687,846 PQSs and their associated *mm%* values (Fig. 3A, Figure S10). The *mm%* distributions were examined for lone PQSs, and separately for those PQSs that have another PQS within their 50-nt flanks. Please note that, as defined above, our PQSs can engulf multiple shorter PQSs, where they are nested or are in a row with no more than 12-nt distance from the prior constituent PQS. To this end, the cases with PQSs in the flanks (Fig. 3A, Figure S10b) are the ones where the inter-PQS distance is greater than 12 nt and shorter than 50 nt (Figure S10). The *mm%* distributions reflected the presence of two distinct clusters, further discussed in Fig. 3A and the description for Figure S10.

For the exploration of the role that the flanking sequences may play in defining the G4 stability in DNA (Fig. 3D), we took advantage of the PQSs subset with the availability of multiple (at least 5) genomic occurrences of the same sequence with different flanks. For such cases, we extracted all the *mm%* values for the individual genomic copies, investigating their mean and standard deviation (Fig. 3D).

### G4 stability of PQSs in the human nuclear genome

Interestingly, against the prior considerations, 50.96% of PQSs do not appear to form stable genomic G4 structures (Fig. 3A, see below for the mapping details), with the remaining 49.04% accounting for the 65.56% of all the G4-seq-observed G4s (525,890 under K^+^/Na^+^ contrasting condition^8^). The rest of the observed G4s were those of non-canonical nature^8^, such as with bulges^36^ and shorter-than-3-nt span for guanines in G-tracts^37,38^. In other words, the usage of the extended PQS motif with 12-nt maximum loop length, though capturing 65.56% of the experimentally observed G4s in the human genome, contained inherent 50.96% false positives (see above). However, the use of the more conservative PQS definition with 7-nt maximum loop-size captured only 36.86% of observed G4s (instead of 65.56%), but with not-much-different, 46.37% fraction of false positives. Therefore, the preferred general motif to hunt for G4s is PQS with 12-nt maximum loop length. However, a human sequence conforming that motif would still have only a 49.04% chance of forming a stable genomic G4.

### Biophysical studies on sequences that comply with the PQS definition but belong to either weak-G4 or stable-G4 clusters of G4-seq

To biophysically interrogate the observed G4 stability differences within PQS motifs, DNA oligonucleotides were randomly picked from PQSs belonging to different ranges of experimental *mm%* values (Table S1). The minimal PQSs (without flanks) were used due to technical difficulties in using longer sequences in standard biophysical experiments. Samples for CD and UV analyses were prepared at 10 μM in a buffer containing 10 mM PBS (pH 7.4) and 100 mM potassium chloride and were annealed prior to each measurement by heating at 95 °C for 5 min and then allowing to cool to 4 °C overnight. CD spectra were recorded on an Applied Photophysics Chirascan Plus circular dichroism spectropolarimeter, using a 1 mm path length quartz cuvette. CD measurements (Fig. 3B,C, Figure S11) were performed at 293 K over a range of 220–320 nm, using a sampling time of 1 s, 1 nm pitch and 0.5 nm bandwidth. The reported spectra represent smoothed averages of three scans that were zero-corrected at 320 nm. Data were recorded as molar ellipticity (×10^5^ deg·cm^2^·dmol^−1^). For UV melting experiments (Table S1, Figure S13), measurements were collected using a Varian Cary 100-Bio UV−visible spectrophotometer by following absorbance at 295 nm. Samples (200 μl) were measured in black, small window, 1 cm path-length quartz cuvettes, covered with a layer of mineral oil (50 μl). Samples were equilibrated at 5 °C for 10 min, heated to 95 °C and cooled back to 5 °C at a rate of 0.5 °C/min. The samples were held for a further 10 min and then the 5 °C to 95 °C ramp was repeated. Data were recorded every 1 °C during both the melting and cooling steps. T_m_ values were obtained from the minimal of the first derivative of the melting curve.^1^H NMR spectra (Figure S12) were recorded at 298 K using a 500 MHz Bruker Avance TCI spectrometer equipped with a cryogenic TCI ATM probe. Water suppression was achieved using excitation sculpting. Samples were annealed at 100 μM in a buffer containing 10 mM PBS (pH 7.4), 100 mM potassium chloride and 10% D_2_O. Samples were analysed and processed using TopSpin software. DNA oligonucleotides used to elucidate the role of flanks in modulating G4 stability (Table S2, Figure S14) were used at 80 μM in the same buffer. The results are discussed in the main text and the referenced figure captions.

### The employed machine learning technique

Tree-based^25^ gradient boosting machine (GBM)^21,23,39^ was used for the machine-learning workflow in our study. We used the eXtreem Gradient Boosting^23^ implementation of GBM (http://github.com/dmlc/xgboost) available in *R* through its *xgboost* library.

### Data pre-processing and feature generation for machine learning

687,846 PQSs, each with its individual G4-seq *mm%* value, were randomly partitioned into 70% and 30% for training and pure test sets respectively (Figure S2, detailed in the caption). This was done after the exclusion of 68,564 PQSs that had other PQSs within their flanks. This exclusion eliminated the instances where the *mm%* values were inflated (Figure S10b) due to the additive effect of multiple G4s on G4-seq base mismatch levels; G4-seq has a resolution nearing the 50-nt length used for defining the flanks around a given PQS, hence any other PQS within those flanks may result in a shared and intensified *mm%* peak.

209 features, based only on DNA sequence and later used in machine learning, were then defined and calculated for each PQS (Figure S1b,c). Of those, 201 were the triad (64) and singleton (3) contents of the PQS, 50-nt-long 5′-flank and 50-nt-long 3′-flank sequences (considered separately). Triad contents were retrieved through a sliding window, by using the lexicological ordering algorithm^40^ for the computational efficiency. The sliding window approach was preferred over the binning one, since the former is capable of capturing extra information for the cases where the given triad is present nested from multiple starts (overlapped instances). This way, for instance, the presence of longer G-tracts (such as GGGG) will be reflected in an extra increment to the GGG triad count (2 if GGGG is present, instead of 1 for only GGG).

Initially, we also tried to use higher k-mers, but their contents were often correlated with the triad contents in PQS. Furthermore, higher k-mers substantially increase the number of features (instead of 64 triads, 256 tetrads or 1024 pentads for each considered segment [5′-flank, PQS, 3′flank]), which may result in overfitting and/or slow computer execution of the potential model, making it useless for genomic applications.

5 other features were added to characterise the topology of the PQS motif, by providing its overall length, number of loops (can be more than 3 in our extended PQS definition that allows for nested motifs) and the lengths of only the first, second and third loops (from 5′-end).

Finally, 3 features described the propensities of hairpin formation at the G-quadruplex loops^41^ (hairpin clipping stabilisation of G-quadruplexes) by providing the ViennaRNA ensemble-averaged folding free energies for the first three loop sequences in PQS. Programmatically, this was achieved by first calculating such energies for all the possible $\sum _{i=5}^{12}{4}^{i}=22,369,280$ sequences that are 5–12-nt long (with parameters for DNA), then adding the obtained values through a look-up table in the feature retrieval workflow.

After compiling the complete set of 209 features for all 5′-flank + PQS + 3′-flank sequences in the training data, the values in each feature pool were centred (by subtracting the median) and scaled (dividing by standard deviation). The median and standard deviation values for each feature set (inferred from the training data) were then saved for the later usage as parameters for data pre-processing in the final stand-alone version of the model.

### Model development workflow

For all the optimisation stages of the machine learning model development, our objective was to minimise the root mean squared error (RMSE) of the predictions. In all but the last stage of the machine learning workflow (*vide infra*), we tuned the major learning parameters that define the topology of the underlying trees (interaction depth, number of trees, minimum child weight) and the exact procedure of gradient boosting (learning rate or shrinkage coefficient, bag fraction or subsample ratio)^22,24^. A given GBM architecture (not model) can thus be defined through a specific configuration of those learning parameters. To assess each configuration for its suitability to a given training dataset (problem), we needed our error metric, as a measure of the performance of the model built with a given configuration, to be unbiased by the training dataset. For this purpose, we used repeated (twice) 3-fold cross-validation procedure^24^ (Figure S4). There, the training data was shuffled twice (for two repeats). Next, for each shuffled state, data were partitioned into three sets, and a model was built and tested three times. Each such instance utilised the merger of two datasets (2/3 of training set) with the third one (1/3 of training set) used for internal testing. The three model-building and testing cycles differed by the choice of the three partitions to use for the merger training set and for the test. Therefore, to assess each parameter configuration in such a repeated (twice) 3-fold cross validation procedure, six model training and internal testing rounds were done, each resulting in an individual error metric. We used rmse_i_ of the predicted vs. actual G4-seq *mm%* values as error metric from each constituent case, and described the overall performance of a given parameter configuration through the RMSE value that is the average of the six constituent ones (Figure S4).

The model was developed by first performing a preliminary GBM model generation based on all 209 features and a reasonable initial set of the 5 parameters that tune the GBM architecture (Figure S3, detailed in the caption). The initial model arrived to the RMSE of 8.291 (*mm%*, from the repeated cross-validation process) with 3500 trees. We used that model to assess the relative importance of each of the 209 used features (Figure S3). Feature importance values were directly obtained from the GBM procedure, where it accounts for the number of times a given feature was used for a split in the underlying decision trees, along with the squared improvement achieved in describing the data after such splits^22^. The examination of the relative feature importance led to the reduction of the number of features to only 119 (Figure S5, detailed in the caption).

Next, by using the optimal set of 119 features, grid sampling of the 5 learning parameters was done to optimise the GBM architecture (Figures S6–S8, detailed in the captions). Parameter sampling was done in three cycles, where the first one tried 330 different combinations (Figures S6c) with fixed 0.01 learning rate (shrinkage coefficient), the second one tried 264 similar combinations but with fixed 0.05 learning rate and slightly reduced upper limit for the tree interaction depth, and the third cycle combined the outcomes of the first two cycles to focus on the putatively optimal learning parameter ranges, fixing the learning rate to 0.01 and increasing the tree interaction depth to 14 (Figures S6b,c). Overall, 634 configurations were tested.

### The final model and its intrinsic validation

The optimal architecture of the GBM suitable for our problem was found to be the one employing 2500 gradient boosted trees, each with 14 branching points (interaction depth) and minimum child weight of 65, constructed by using 0.01 shrinkage factor (learning rate) and resampling ratio of 0.6 (bag fraction) (Figure S6). This GBM architecture was then used to train a model using the complete set of training data (433,498, Figure S9), without cross-validation cycles (Figure S4) that were originally leaving out 1/3 of data from each training round during the GBM architecture tuning process. To this end, although the repeated cross-validation cycles (intrinsic validation) resulted in 8.21% RMSE for the most optimal architecture (Figure S6), we expected the performance of the final model to improve even more (lower RMSE, see below), owing to the usage of the extended (e.g. complete training) dataset in the final training.

### Validation of the final model on the external pure test dataset

The final model (Fig. 4E) was then tested on the external test dataset (185,784 entries) that had been left out from the very beginning of the model development, and did not participate in either the GBM architecture tuning or the final model training runs. The model was deemed to be rather reproducible on such a dataset, expressing 8.14% RMSE and high (Pearson’s R = 0.80) correlation with the experimental G4-seq mismatch levels (Fig. 5A). We further determined the *mm%* threshold of classifying the belonging of a given PQS to either the stable G4-forming or non-G4 clusters (Fig. 5B,C), arriving at 19.1% for the best true positive and true negative rate combination (while trying to maximise both) in the simpler classification procedure.

### Feature importance in the final model, along with their directionality estimation

Feature importance is an indirect metric that accounts for the number of times a given feature was used for a split in the decision trees behind the tree-based GBM, along with the squared improvement achieved in describing (fitting) the training data after such splits^22^. The relative importance values were obtained from the GBM procedure as detailed elsewhere^22,24^, and were further normalized for the most influential feature to have a value of 1 (Fig. 5D, or 100 as in Table S3). Hence, for the rest of the features, importance values represent the fractions of the importance from the most influential feature. A crude estimation (discarding complex conditional interdependences) for the most pronounced directionality of each feature, i.e. whether an increase in a given feature value leads to an increase in G4 stability (blue “ + ” in Fig. 5D and Figure S16) or vice versa (red “−” in Fig. 5D and Figure S16), was done as described in Table S3. A green asterisk (“*”) is used for the features that have more complex connection with the *mm%*, not apparent *via* the simplistic means described in Table S3. The feature names should be deciphered as follows: the prefixes G4, 5′f and 3′f denote the features extracted from the PQS, 5′-flank and 3′-flank segments of DNA respectively; the single-letter suffixes denote the singleton contents; three-letter suffixes denote the corresponding triad counts; the suffixes *numlps*, *lp1len*, *lp2len*, *lp3len*, *length*, *lp1efe*, *lp2efe* and *lp3efe* denote the number of loops in the extended PQS definition, length of the first, second and third loops, overall length of PQS, ensemble averaged free energies for the sequences of the first, second and third loops. The full list of all the features with the importance values and crude directionality analysis is brought in Table S3.

### Data availability

Quadron is open source and is freely accessible from http://quadron.atgcdynamics.org. Figures S1–S18, Tables S1–S4 and Supporting References are available in the Supplementary Information.

## Electronic supplementary material

Supplementary Information

## References

[1] Eddy J, Maizels N (2006). Gene function correlates with potential for G4 DNA formation in the human genome. Nucl. Acids Res..

[2] Bochman ML, Paeschke K, Zakian VA (2012). DNA secondary structures: stability and function of G-quadruplex structures. Nat. Rev. Genet..

[3] Biffi G, Tannahill D, McCafferty J, Balasubramanian S (2013). Quantitative visualization of DNA G-quadruplex structures in human cells. Nat. Chem..

[4] Hänsel-Hertsch R (2016). G-quadruplex structures mark human regulatory chromatin. Nat. Genet..

[5] Sahakyan AB, Murat P, Mayer C, Balasubramanian S (2017). G-quadruplex structures within the 3′ UTR of LINE-1 elements stimulate retrotransposition. Nat. Struct. Mol. Biol..

[6] Maizels N (2008). Genomic stability: FANCJ-dependent G4 DNA repair. Curr. Biol..

[7] Adrian M, Heddi B, Phan AT (2012). NMR spectroscopy of G-quadruplexes. Methods.

[8] Chambers VS (2015). High-throughput sequencing of DNA G-quadruplex structures in the human genome. Nat. Biotech..

[9] Huppert J, Balasubramanian S (2005). Prevalence of quadruplexes in the human genome. Nucl. Acids Res..

[10] Todd AK, Johnston M, Neidle S (2005). Highly prevalent putative quadruplex sequence motifs in humanDNA. Nucl. Acids Res..

[11] Maizels N, Gray LT (2013). The G4 genome. PLoS Genet..

[12] Kikin O, D’Antonio L, Bagga PS (2006). QGRS Mapper: a web-based server for predicting G-quadruplexes in nucleotide sequences. Nucl. Acids Res..

[13] Stegle O, Payet L, Mergny J-L, MacKay DJC, Leon JH (2009). Predicting and understanding the stability of G-quadruplexes. Bioinformatics.

[14] Lorenz R (2013). 2D meets 4G: G-quadruplexes in RNA secondary structure prediction. IEEE Trans. Comput. Biol. Bioinform..

[15] Yano M, Kato Y (2014). Using hidden Markov models to investigate G-quadruplex motifs in genomic sequences. BMC Genomics.

[16] Bedrat A, Lacroix L, Mergny J-L (2016). Re-evaluation of G-quadruplex propensity with G4Hunter. Nucl. Acids Res..

[17] Alipanahi B, Delong A, Weirauch MT, Frey BJ (2015). Predicting the sequence specificities of DNA- and RNA-binding proteins by deep learning. Nat. Biotech..

[18] Whitaker JW, Chen Z, Wang W (2015). Predicting the human epigenome from DNA motifs. Nat. Meth..

[19] Leung MKK, Delong A, Alipanahi B, Frey BJ (2016). Machine learning in genomic medicine: a review of computational problems and data sets. Proceed. IEEE.

[20] Arora A, Nair DR, Maiti S (2009). Effect of flanking bases on quadruplex stability and Watson-Crick duplex competition. FEBS J..

[21] Friedman, J. H. Greedy function approximation: a gradient boosting machine. *IMS Reitz Lecture* 1–39, accessible from http://statweb.stanford.edu/~jhf/ftp/trebst.pdf (1999).

[22] Natekin A, Knoll A (2013). Gradient boosting machines, a tutorial. Front. Neurorobot..

[23] Chen, T. & Guestrin, C. XGBoost: a scalable tree boosting system. *arXi*v **1603.02754v3**, 1–13 (2016).

[24] Kuhn, M. & Johnson, K. *Applied predictive modeling*. (Springer, 2013).

[25] Hastie, T., Tibshirani, R. & Friedman, J. H. *10. Boosting and additive trees*. 337–387 (Springer, 2009).

[26] Caruana, R. & Niculescu-Mizil, A. An empirical comparison of supervised learning algorithms. in 161–168, 10.1145/1143844.1143865 (ACM Press, 2006).

[27] Godfrey, J. Using boosted decision trees for tau identification in the ATLAS experiment. 1–119, a thesis accessible from http://cds.cern.ch/record/2244641 (2009).

[28] Lin J (2013). Stabilization of G-quadruplex DNA by C-5-methyl-cytosine in bcl-2 promoter: implications for epigenetic regulation. Biochem. Biophys. Res. Comm..

[29] R Core Team. R:a language and environment for statistical computing. *R Foundation for Statistical Computing, Vienna, Austria* (2015).

[30] Xiong HY (2015). The human splicing code reveals new insights into the genetic determinants of disease. Science.

[31] Libbrecht MW, Noble WS (2015). Machine learning applications in genetics and genomics. Nat. Rev. Genet..

[32] Costantini M, Clay O, Auletta F, Bernardi G (2006). An isochore map of human chromosomes. Genome Res..

[33] Guédin A, Gros J, Alberti P, Mergny J-L (2010). How long is too long? Effects of loop size on G-quadruplex stability. Nucl. Acids Res..

[34] Agrawal P, Lin C, Mathad RI, Carver M, Yang D (2014). The major G-quadruplex formed in the human BCL-2 proximal promoter adopts a parallel structure with a 13-nt loop in K^+^ solution. J. Am. Chem. Soc..

[35] Jodoin R (2014). The folding of 5′-UTR human G-quadruplexes possessing a long central loop. RNA.

[36] Mukundan VT, Phan AT (2013). Bulges in G-quadruplexes: broadening the definition of G-quadruplex-forming sequences. J. Am. Chem. Soc..

[37] Phan AT, Kuryavyi V, Luu KN, Patel DJ (2007). Structure of two intramolecular G-quadruplexes formed by natural human telomere sequences in K^+^ solution. Nucl. Acids Res..

[38] Li X-M (2015). Guanine-vacancy-bearing G-quadruplexes responsive to guanine derivatives. Proc. Natl. Acad. Sci. USA.

[39] Friedman, J. H. Stochastic gradient boosting. 1–10, accessible from http://statweb.stanford.edu/~jhf/ftp/stobst.pdf (1999).

[40] Compeau, P. & Pevzner, P. *Bioinformatics algorithms: an active learning approach*. (Active Learning Publishers, 2014).

[41] Lim KW (2015). Duplex stem-loop-containing quadruplex motifs in the human genome: a combined genomic and structural study. Nucl. Acids Res..

[42] Paramasivan S, Rujan I, Bolton PH (2007). Circular dichroism of quadruplex DNAs: applications to structure, cation effects and ligand binding. Methods.

[43] Masiero S (2010). A non-empirical chromophoric interpretation of CD spectra of DNA G-quadruplex structures. Org. Biomol. Chem..

