# Supplementary material for: Machine learning model for sequence-driven DNA G-quadruplex formation
Source: Sci Rep. 2017 Nov 6;7:14535. doi: 10.1038/s41598-017-14017-4 (PMC5673958; doi:10.1038/s41598-017-14017-4)
Supplement: Supplementary file 1 — Supplementary Information [file 41598_2017_14017_MOESM1_ESM.pdf]

SUPPORTING INFORMATION

Machine learning model for sequence-driven DNA G-quadruplex formation

Aleksandr B. Sahakyan,[1,‡] Vicki S. Chambers,[1,‡] Giovanni Marsico,[1,2] Tobias Santner,[1] Marco Di Antonio,[1,2] and Shankar Balasubramanian[1,2,3,*]

[1] Department of Chemistry, University of Cambridge, Lensfield Road, Cambridge CB2 1EW, UK. [2] Cancer Research UK Cambridge Institute, University of Cambridge, Li Ka Shing Centre, Robinson Way, Cambridge CB2 0RE, UK. [3] School of Clinical Medicine, University of Cambridge, Cambridge CB2 0SP, UK. [‡] These authors contributed equally to the work. [*] Correspondence to Prof. Shankar Balasubramanian (sb10031@cam.ac.uk).

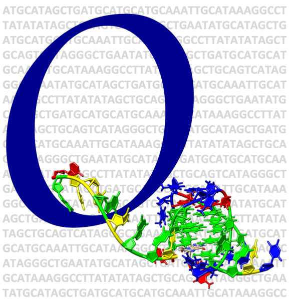

CONTENTS:

Figure S1 •••••••••••••••••••••••••••••• 2
Figure S2 •••••••••••••••••••••••••••••• 3
Figure S3 •••••••••••••••••••••••••••••• 4
Figure S4 •••••••••••••••••••••••••••••• 5
Figure S5 •••••••••••••••••••••••••••••• 6
Figure S6 •••••••••••••••••••••••••••••• 7
Figure S7 •••••••••••••••••••••••••••••• 8
Figure S8 •••••••••••••••••••••••••••••• 9
Figure S9 •••••••••••••••••••••••••••••• 10
Figure S10 •••••••••••••••••••••••••••••• 11
Figure S11 •••••••••••••••••••••••••••••• 12
Figure S12 •••••••••••••••••••••••••••••• 13
Figure S13 •••••••••••••••••••••••••••••• 14
Figure S14 •••••••••••••••••••••••••••••• 15
Figure S15 •••••••••••••••••••••••••••••• 16
Figure S16 •••••••••••••••••••••••••••••• 17
Figure S17 •••••••••••••••••••••••••••••• 18
Figure S18 •••••••••••••••••••••••••••••• 19
Table S1 •••••••••••••••••••••••••••••• 20
Table S2 •••••••••••••••••••••••••••••• 21
Table S3 •••••••••••••••••••••••••••••• 22
Table S4 •••••••••••••••••••••••••••••• 25
Supporting References •••••••••••••••••••• 26

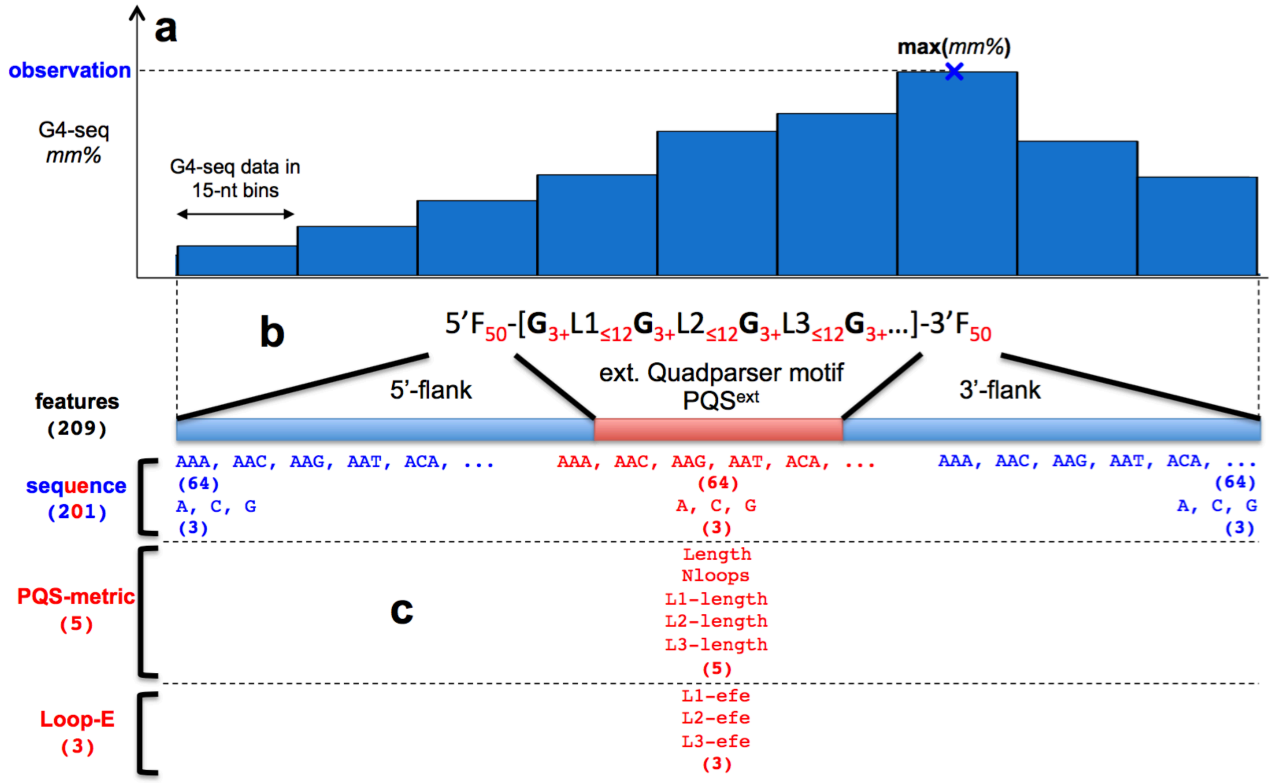

Figure S1 | Selection of features and a single individual G4-seq *mm%* value per putative quadruplex sequence. Machine-supervised learning procedure requires training data, where each entry contains both the necessary response value, and the set of features to build the model upon. As a response value, we used the maximum experimental G4-seq *mm%* (labelled with blue × in example **a**) observed among all the *mm%* values, measured for each 15-nt bin that overlap with the given extended PQS sequence and its 50-nt long 5'- and 3'-flanks (see **a** and **b**). The number of 15-nt bins that overlap with PQS+flanks can vary depending on the length of the PQS. We then defined 209 features fully based on the DNA sequence of PQS and its flanks (**c**). Of those, 201 were the triad (64) and singleton (3) contents of the PQS, 50-nt-long 5'-flank and 50-nt-long 3'-flank sequences (considered separately). 5 were describing the overall architecture of the quadruplex sequence and 3 were reflecting the stem-loop-formation ensemble averaged free energies of the first three loop sequences of PQS (**c**). For complete details, see subsection **11** of **Methods**.

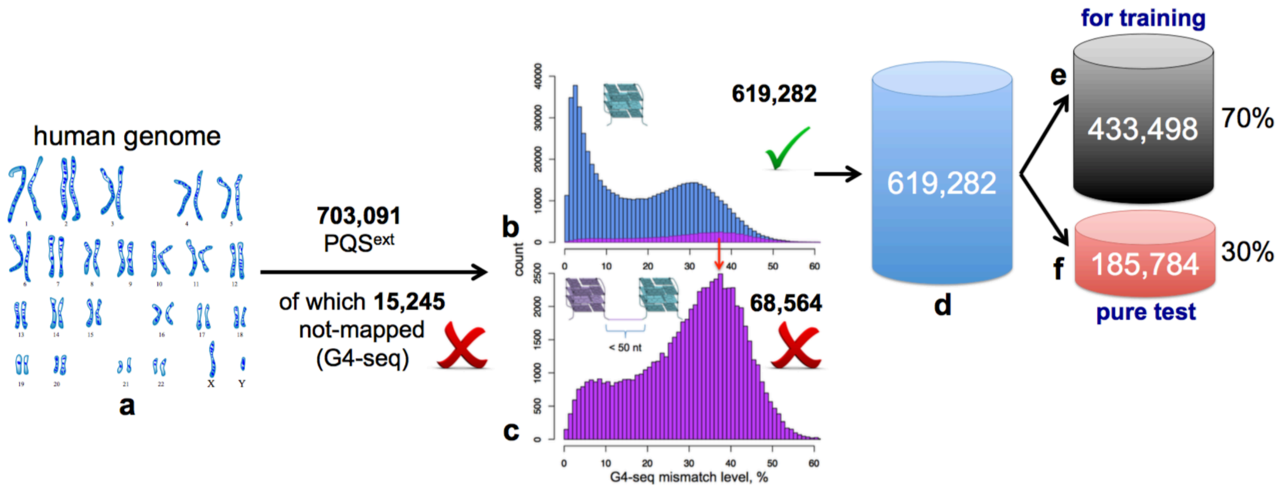

Figure S2 | Data partitioning for machine learning. The application of the extended Quadparser sequence motif to search for putative quadruplex sequences (PQS) throughout the human genome produced 703,091 hits, of which 15,245 were not mapped in the G4-seq experiment, hence were left out. From the remainder, 619,282 sequences (**b**) were further considered (along with their 50-nt flanks in both 5’ and 3’ directions), since the other 68,564 contained additional PQSs within their flanks (**c**). The latter exclusion was necessary due the additive effect reflected in G4-seq *mm%* values where there were multiple G4s immediately following each other (**c**), which normally resulted in overestimated *mm%* values assigned to the constituent G4s in such clusters (**Figure S10**). The 619,282 sequences (**d**) were next randomised and partitioned to 433,498 (70%) for training purposes (**e**) and 185,784 (30%) for pure testing (**f**). During the architecture adjustment stage of the machine learning workflow based on gradient boosting machine (GBM)[1-3], the training dataset was subsampled to produce random test sets during the repeated cross-validation process (see below, **Figure S4**). The error metrics obtained from such subsampled test sets, though not directly involved in the training process, are still used for the decision making in the selection of the best GBM architecture, hence indirectly take part in the machine learning procedure. It is therefore of paramount importance to leave out a “pure” dataset from the very beginning, where the data have no direct and indirect relation to the machine learning process. Pure test in **f** was thus created to achieve just that, with the absence of the underlying data from both the model parameterisation and the optimisation of the machine learning architecture (learning parameters in GBM). Note that both training and pure test datasets held PQSs with the high-to-low (> 18 *mm%* vs. < 18 *mm%*) ratio of actual G4 formation propensities in G4-seq experiments being 1.000/1.039, thus devoid of a imbalanced data representation and associated danger of biasing our outcomes.

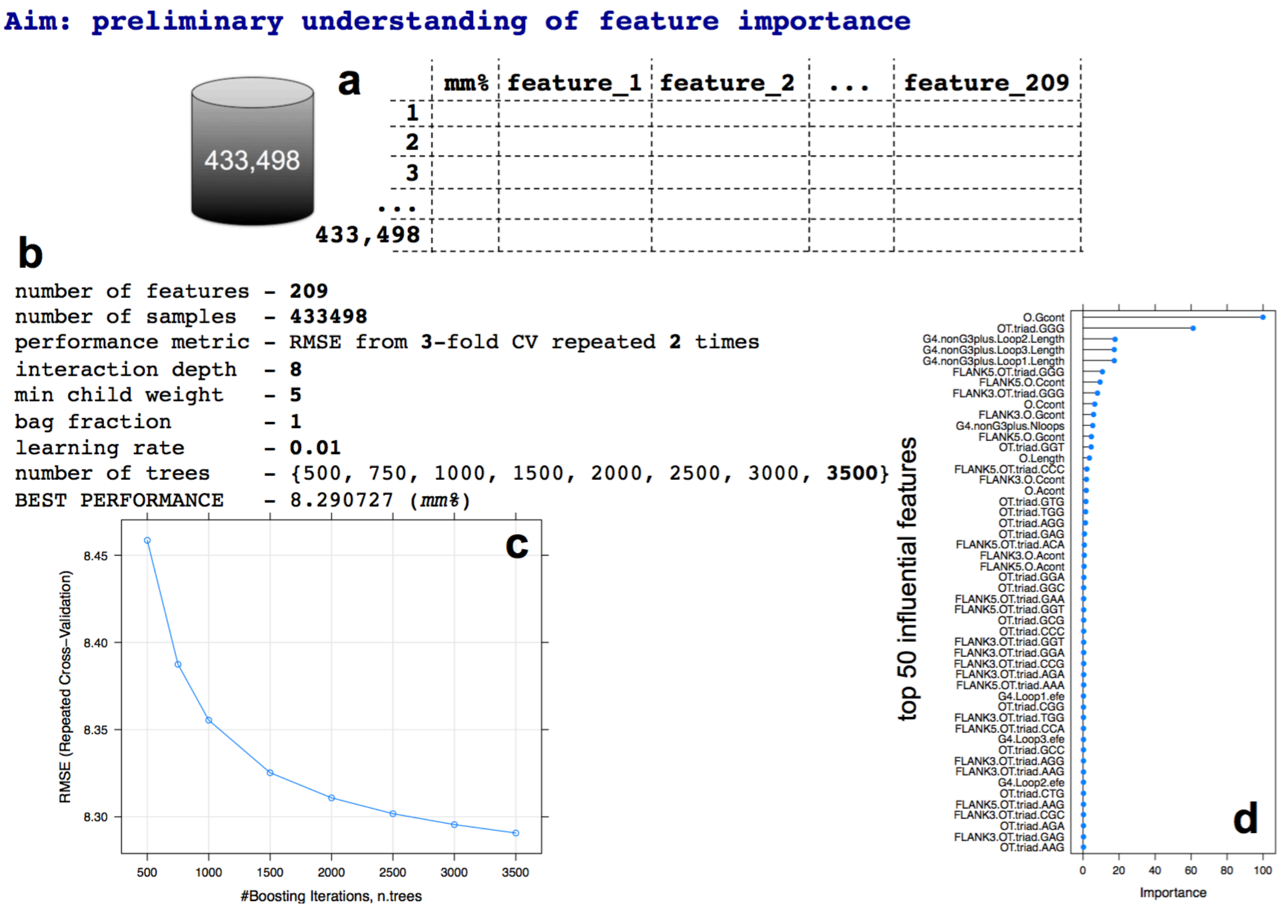

Figure S3 | Preliminary GBM model generation for the initial assessment of feature importance. For the first stage of the machine learning workflow (see **Methods**), we have utilised all the 209 generated features (**a**), and parameterised a preliminary model by using a reasonably sophisticated (low learning rate, high interaction depth and maximum number of trees, **b**) set of learning parameters and optimising only the number of trees (**b**). In the notations in **b**, the values within the curly brackets denote the sampled numbers of trees. The initial model arrived to the RMSE of 8.291 (*mm%*, from the repeated cross-validation process) with 3500 trees (**c**). We used that model to assess the relative importance of each of the 209 used features (**d**, example relative importance of the top 50 influential features), normalising the values by setting the relative importance of the most influential feature to 100. Feature importance values were directly obtained from the GBM procedure, where it accounts for the number of times a given feature was used for a split in the underlying decision trees, along with the squared improvement achieved in describing the data after such splits[3].

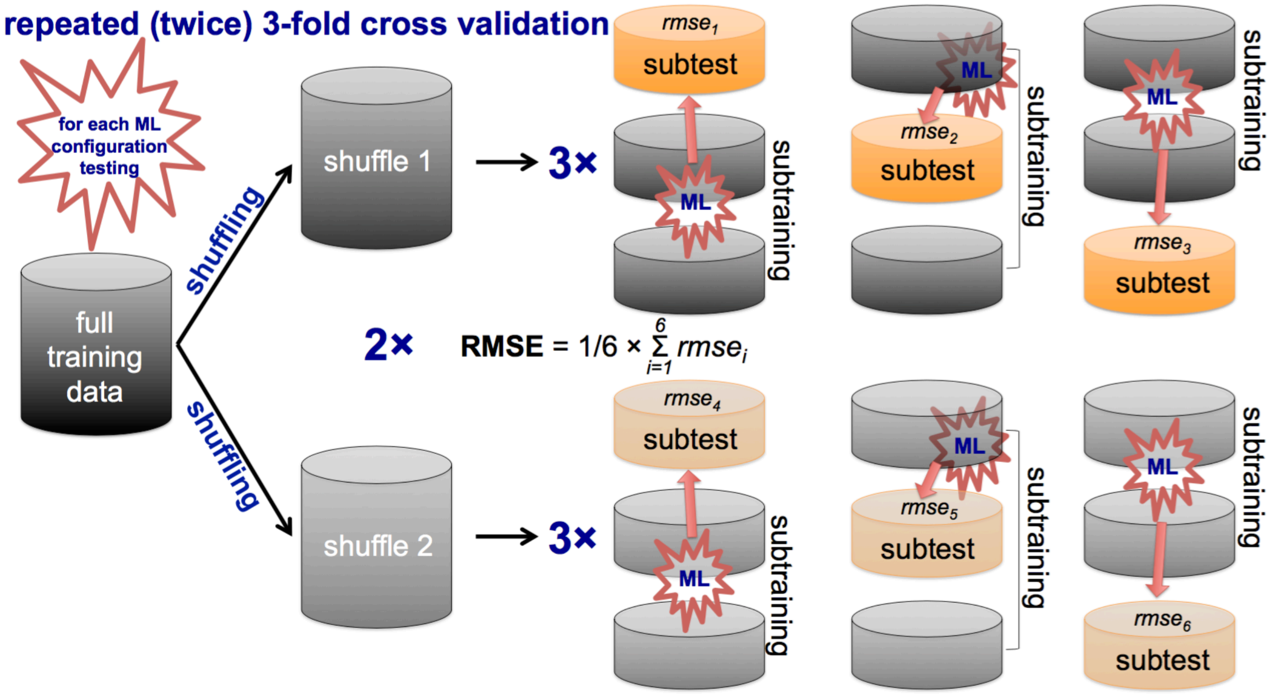

Figure S4 | Repeated cross-validation procedure and error metric used within GBM architecture tuning stages. In all but the last stage of the machine learning workflow, we tuned the major learning parameters that define the topology of the underlying trees (interaction depth, number of trees, minimum child weight) and the exact procedure of gradient boosting (learning rate or shrinkage coefficient, bag fraction or subsample ratio)[2,3]. A given GBM architecture can thus be defined through a specific configuration of those learning parameters. To assess each configuration for its suitability to a given dataset (problem), we need an unbiased error metric to measure the performance of the model built with such a configuration on the training data. For this purpose, we used repeated (twice) 3-fold cross-validation procedure[2]. There, the training data is shuffled twice (for two repeats). Next, for each shuffled state, data is partitioned into three sets, and a model is built and tested three times. Each such instance utilised the merger of two datasets (2/3 of training set) with the third one (1/3 of training set) used for testing. The three model-building and testing cycles differ by the choice of the three partitions to use for the merger training set and for the test. Therefore, to assess each parameter configuration in such a repeated (twice) 3-fold cross validation procedure, six model training and internal testing rounds is done, each resulting in an individual error metric. We used root mean squared deviation ($rmse_i$) of the predicted vs. actual G4-seq *mm%* values as error metric from each constituent case, and described the overall performance of a given parameter configuration through the RMSE value that is the average of the six constituent ones.

Aim: feature optimisation

a

```
number of features - 209 (all), 119 (> 0.1 vimp[100]), 60 (> 0.2), 32 (> 0.3)
number of samples  - 433498
performance metric - RMSE from 3-fold CV repeated 2 times
interaction depth  - 8
min child weight   - 5
bag fraction       - 1
learning rate      - {0.1, 0.01}
number of trees    - {500, 750, 1000, 1500, 2000, 2500, 3000, 3500}
BEST PERFORMANCE   - 8.288949 (mm%)
```

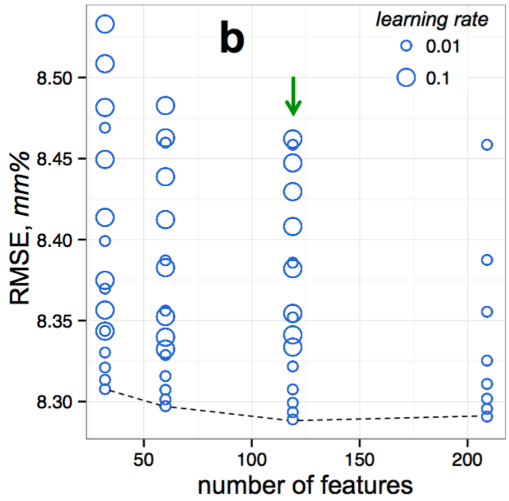

Result: we can eliminate 90 features without any loss (even with a slight gain) in performance.

Figure S5 | Optimisation of the number of features used in the GBM model development. Based on the preliminary GBM model and the crude relative importance estimation of all generated 209 features in that model, we next (2nd stage) explored how many features can be excluded without influencing the GBM model performance. We generated models with varying GBM architectures for four classes of models, using all 209, the top 119 (removing all the features that are more than 1000 times weaker than the most influential feature), the top 60 (removing all the features that are more than 300 times weaker than the most influential feature) and the top 32 (removing all the features that are more than 200 times weaker than the most influential feature) features (**a**). For each class, we additionally tried 2 different learning rates (shrinkage coefficients) and 8 different values for the number of trees. We set the rest of the learning parameters to define the GBM architecture equal to the values in the initial model (**Figure S3**). In the notations in **a**, the numbers within the curly brackets denote the sampled values for each corresponding learning parameter, where all the possible permutations of those values across the 2 optimised parameters (learning rate and number of trees) were sampled. At this stage, we learned that 90 features could be eliminated without any loss in the performance of GBM models. Such elimination left only the top 119 features (**b**) to be used for the further optimisation of the GBM architecture, which increased the computation speed and reduced the memory usage at all levels of further model development.

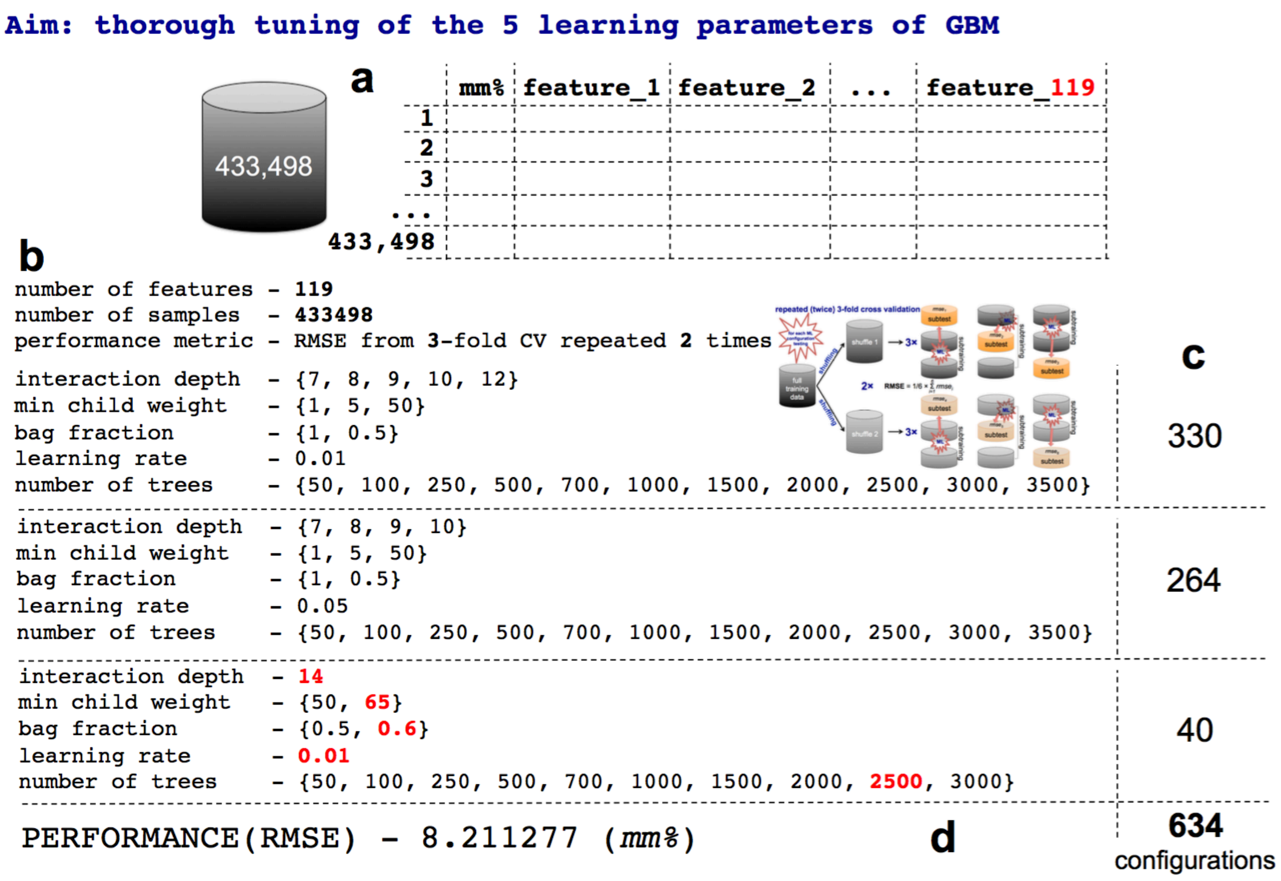

Figure S6 | Tuning the GBM architecture. At the 3rd stage of the machine learning workflow, we started from the training dataset and the optimally reduced 119 features (**a**) to try different values for 5 learning parameters (**b**), with the aim to find the optimal combination. For each parameter configuration, the model was built and tested using a repeated (twice) 3-fold cross-validation process, employing RMSE as the performance metric. Parameter sampling was done in three cycles, where the first one tried 330 different combinations (**c**) with fixed 0.01 learning rate (shrinkage coefficient), the second one tried 264 similar combinations but with fixed 0.05 learning rate and slightly reduced upper limit for the tree interaction depth, and the third cycle combined the outcomes of the first two cycles to focus on the putatively optimal learning parameter ranges, fixing the learning rate to 0.01 and increasing the tree interaction depth to 14 (**b**, **c**). We did not try interaction depths greater than 14, taking into account the substantial compromise in computational speed that may prevent the genome-wide applicability of any outcome model. In the notations in **b**, the numbers within the curly brackets denote all the sampled values for each corresponding learning parameter, where all the possible permutations (with the overall number indicated at the right side) of those values across the 5 parameters were sampled. The found optimal values (at the third cycle) are highlighted in red, with the overall RMSE from the cross-validation process settling at 8.21 (*mm%*) after trying overall 634 configurations (**d**).

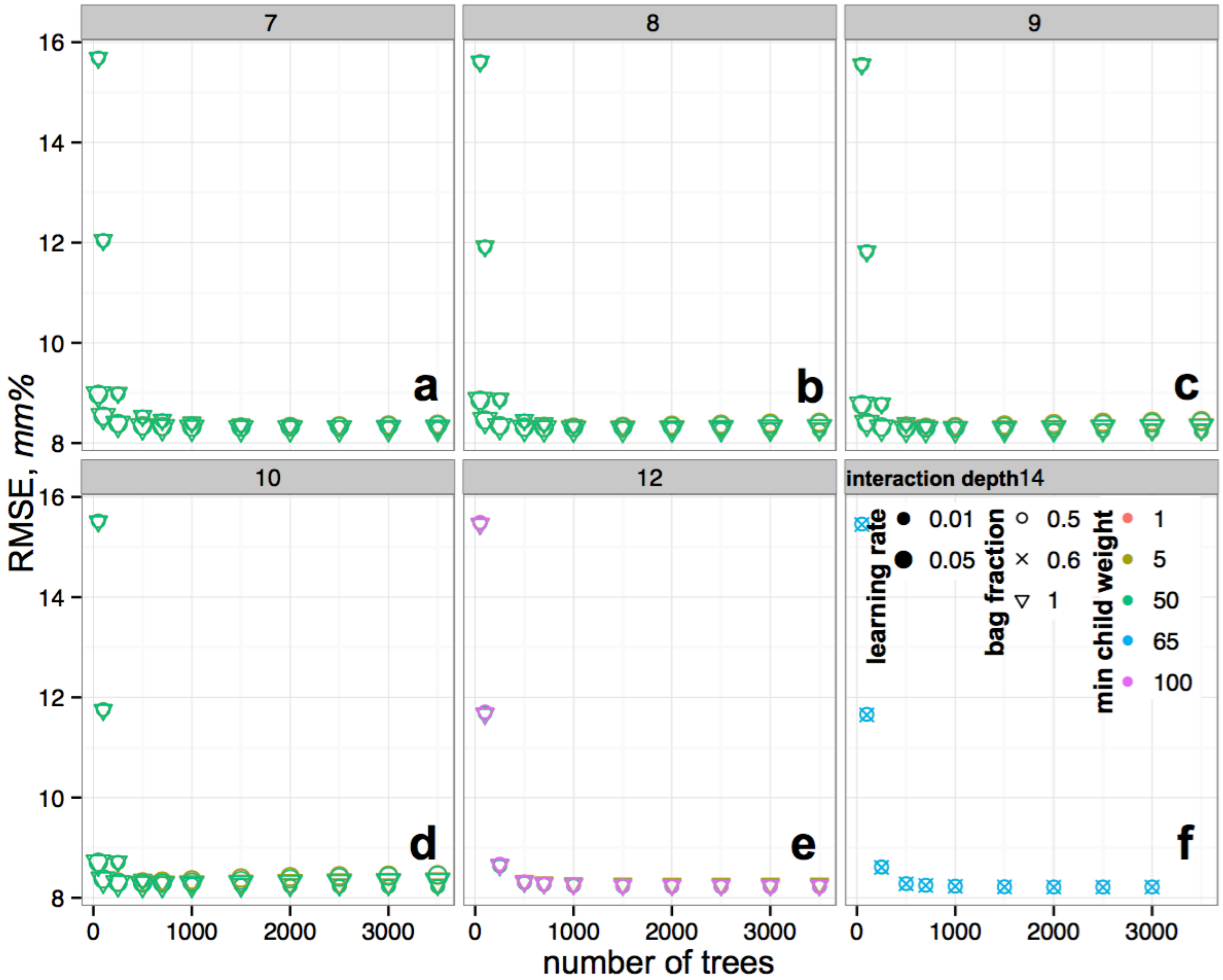

Figure S7 | RMSE variation across different GBM learning parameter configurations. The plots **a-f** correspond to different values of interaction depth for the tree complexity, as indicated on top of each plot. The x-axes capture the variation in the number of trees, while the size, shape and colour of the individual points denote the learning rate (shrinkage coefficient), bag (subsampling) fraction and minimum child weight, of the learning parameters[2,3], with the key indicated in **f**. The plots demonstrate a rapid convergence toward a reasonable range of errors (low RMSE) for the models, as soon as 500 and more trees are used. The details of the lower RMSE region are visible in the zoomed version of the figure shown below.

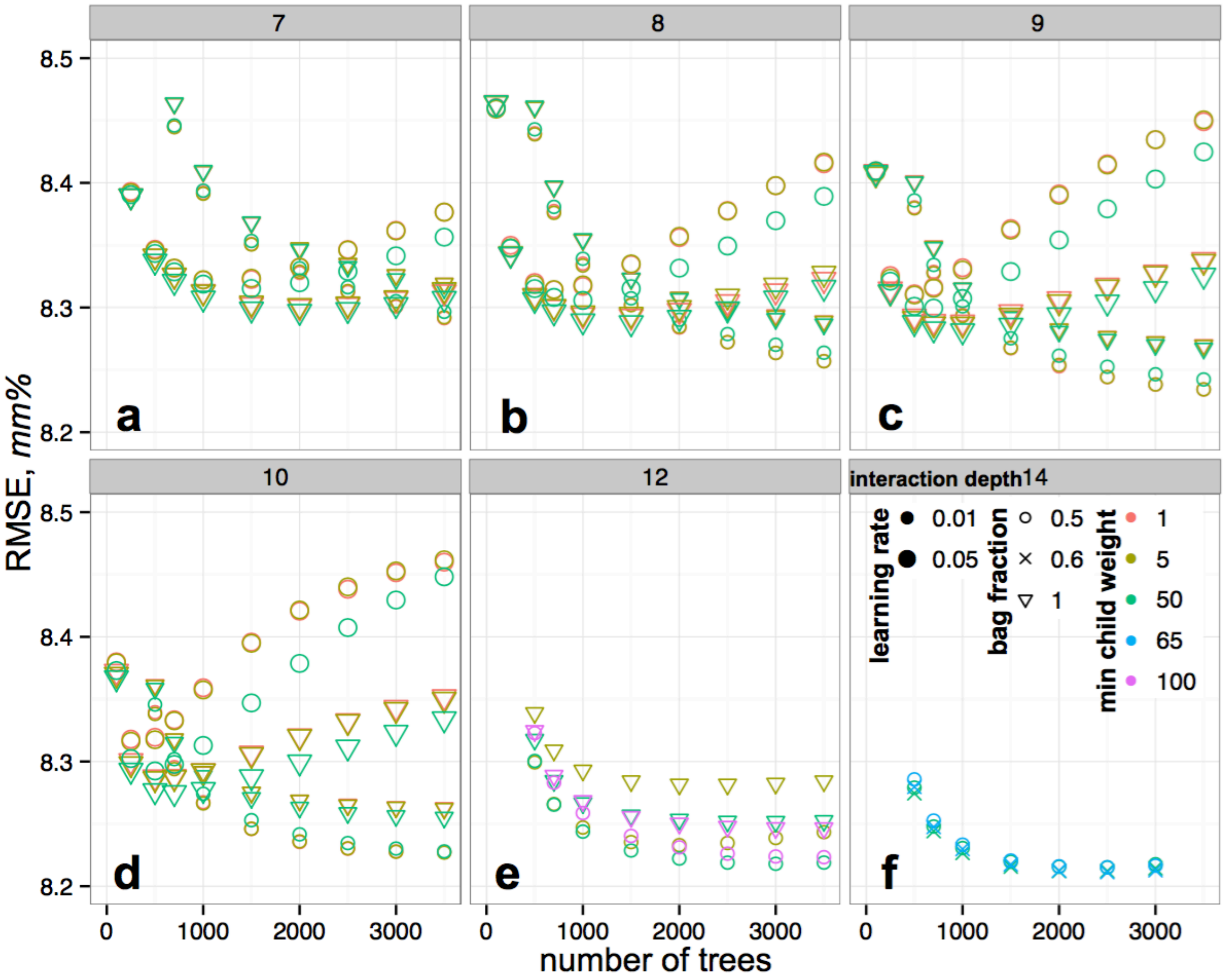

Figure S8 | RMSE variation across different GBM learning parameter configurations: the zoomed plots. The plots **a-f** correspond to different values of interaction depth for the tree complexity, as indicated on top of each plot. The x-axes capture the variation in the number of trees, while the size, shape and colour of the individual points denote the learning rate (shrinkage coefficient), bag (subsampling) fraction and minimum child weight, of the learning parameters[2,3], with the key indicated in **f**. The plots demonstrate the preference towards the configurations with lower learning rate and higher number of trees in the case of our particular problem. The data shown are identical to **Figure S7**, but with y-axes zoomed (range 8.2-8.5) to better visualise regions of lower RMSE.

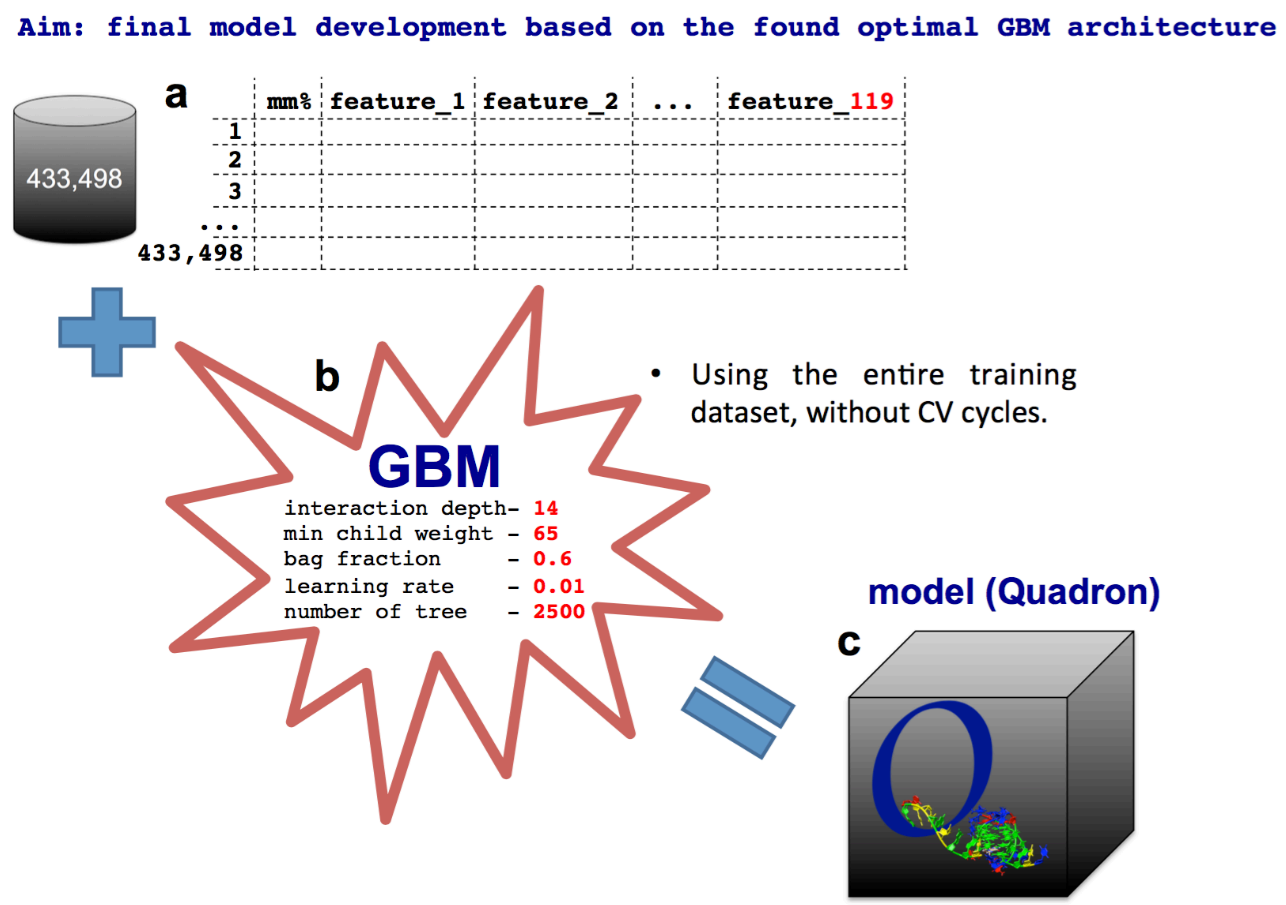

Figure S9 | Final Quadron model generation using the entire training dataset. This 4th stage was based on the previously revealed 119 features (**a**) and the set of 5 optimal parameters defining the GBM architecture (**b**). The final model was then built in a single run, utilising the complete set of training data (433,498 entries), without any cross-validation cycles, hence without part of the data internally excluded to be used as internal tests. The model (**c**) was produced as an *R*[4] object that works with the xgboost library (http://github.com/dmlc/xgboost).

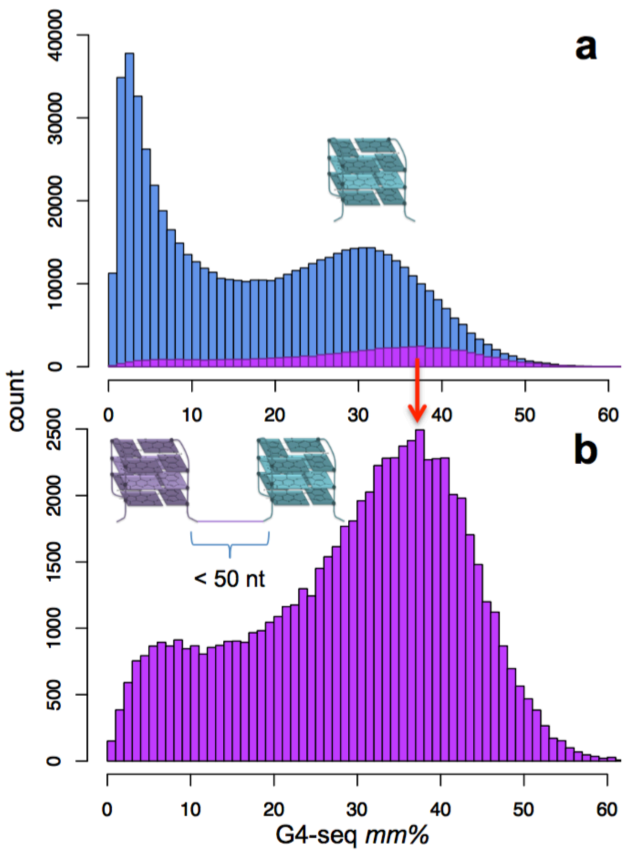

Figure S10 | Distribution of G4-seq ***mm%*** **for all the extended putative quadruplex sequences (PQS).** The PQS motif was used as defined in **Methods**, with 1) maximum loop size of 12 nt[5]; 2) allowing nested G4s, if the additional G-tracts are present not apart from the 4th G-tract by more than the maximum loop size (12 nt). The plots illustrate the presence of two clusters, where the sequences with low *mm%* represent low stability genomic G4-structures, as compared to the sequences with higher *mm%*[6]. Two cases of the *mm%* distribution were considered colored in blue and magenta (histograms **a** and **b**). In contrast to the blue one (**a**), the magenta histogram (**a**, additionally zoomed in **b**) depicts PQSs with another PQS present overlapping with 50-nt flanks. Such cases are prone to present inflated *mm%* values owing to the additive accumulation of base mismatches from two G4 structures under the G4-seq condition. The shift toward the higher *mm%* values for the "not-lone" PQSs is reflected in **b**. Such sequences were later eliminated from the model building, owing to their relative sparseness and the necessity for the model to reflect the correct formation propensity for individual G4s, regardless the presence of another G4 nearby.

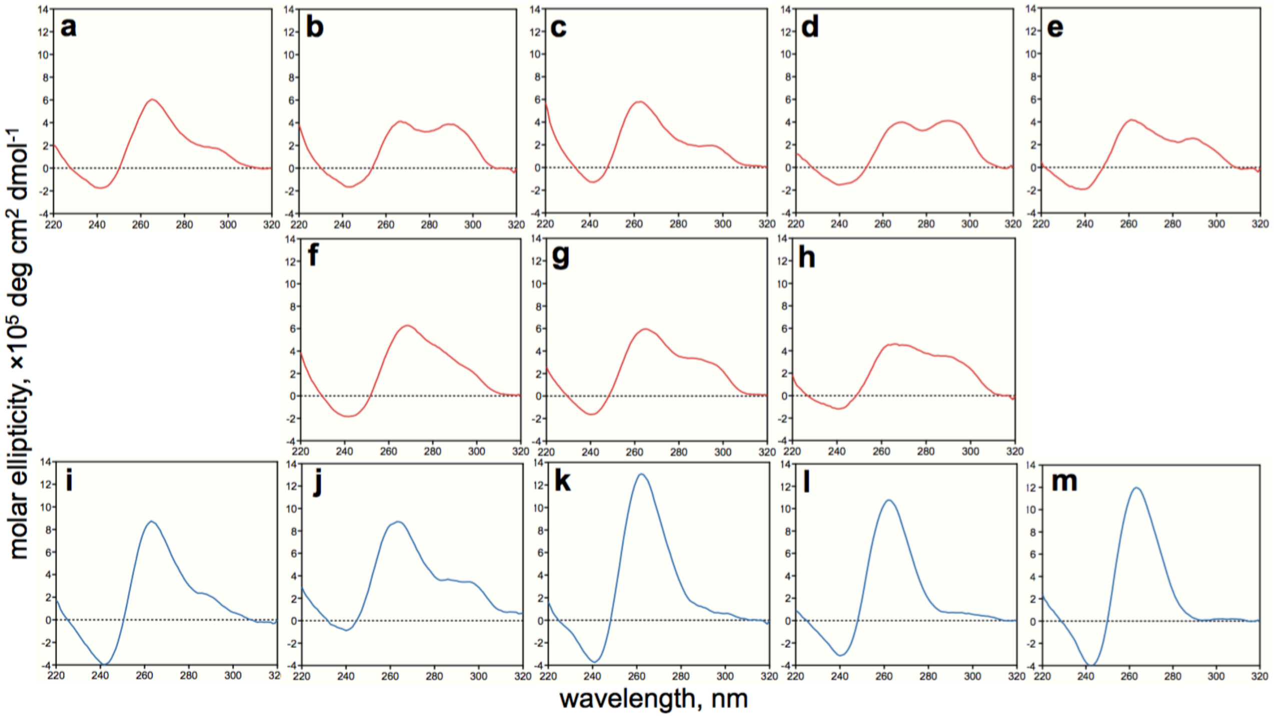

Figure S11 | Circular dichroism (CD) characterisation of sequences with different G4-seq *mm%* levels. The CD experiments were done on the PQSs only (**Table S1**), without the genomic context that is in the G4-seq experiment[6]. Typical G4 signatures[7] with a maximum at ~260 nm and a minimum at ~240 nm for parallel topology, or a maximum at ~290 nm and a minimum at ~260 nm, for antiparallel topology, are appreciable to a different extent for all the sequences tested, however, with more pronounced and intense G4 signatures for the sequences **i**-**m** (blue spectra, as compared to red spectra for sequences **a**-**h**) that form stable genomic G4 structures (> 18 *mm%*) in G4-seq[6].

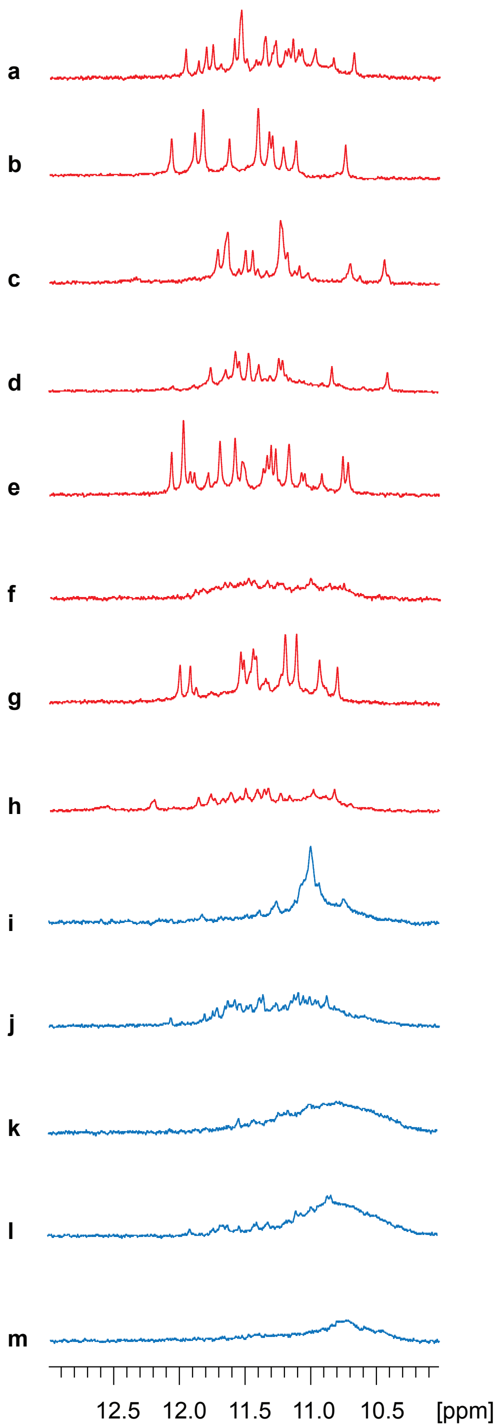

Figure S12 | NMR characterisation of sequences with different G4-seq *mm%* levels. ^{1}H NMR spectra revealed proton peaks[8] associated with Hoogsteen base-paired guanines in G4s (10-12 ppm) for all the sequences investigated. However, the sequences **i-m** (blue spectra, as compared to red spectra for sequences **a-h**, **Table S1**) that formed stable genomic G4 structures (> 18 *mm%* in G4-seq[6]) were distinct in having broader merged signals, reflecting either the presence of multiple conformers or highly ordered quadruplex cores[9], both contributing to the increased stability. Taken together with G4-seq data, CD spectra (**Figure S11**) and UV melting experiments (**Figure S12**, **Table S1**), where the melting temperatures correlated well with the *mm%* hierarchy observed through G4-seq, the data lead to the conclusion that the weak-G4 cluster in **Figure 3A** indeed contained sequences that either did not form G4s in a genomic context, or formed rather unstable ones.

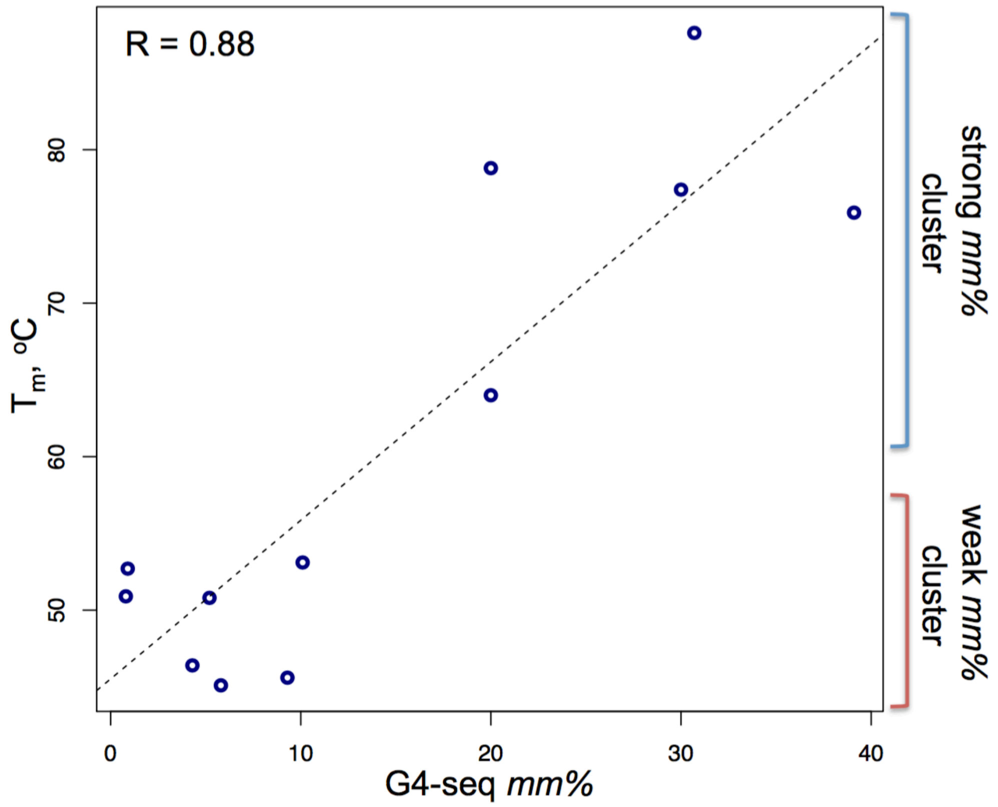

Figure S13 | UV-spectroscopy determined melting temperatures of the studied sequences compared to mismatch levels measured by G4-seq. The G4 structure melting temperatures (T_m) were obtained from the minimum of the first derivative of the melting curves while following the absorbance at 295 nm[10]. The values reflected in the plot are summarised in **Table S1**. Despite the different nature and context of the short-sequence-based UV spectroscopy and genomic G4-seq experiments, a reasonable (Pearson's R = 0.88) correlation can still be noted between the T_m and *mm%* values. This demonstrates that the G4-seq mismatch levels correlate with the stability of G4 structures, even though, unlike the UV data, G4-seq reports upon such stabilities in the context of the genomic DNA[6]. The linear trendline is shown on the plot as a dashed line.

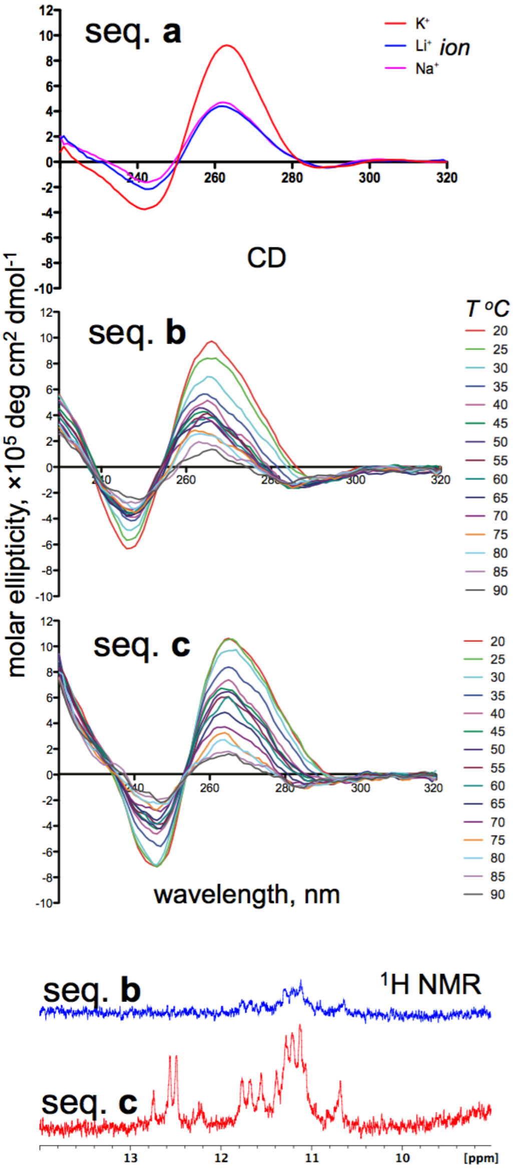

Figure S14 | NMR and CD investigation of the effect generated by different flanking sequences in G4 formation propensity. A G4 sequence (sequence **a** in **Table S2**) was selected, which, depending on the flanks (50-nt long from both sides), showed low (7.4, sequence **b** in **Table S2**) and high (45.7, sequence **c** in **Table S2**) G4-seq *mm%* values. First, a CD characterization was done for the sequence **a** without the flanks, in G4-stabilising (K^+) and contrasting (Na^+, Li^+) conditions. The biophysical experiments for the longer sequences **b** and **c** were complicated by the length (115 nt) of the sequences, with no clear melting points differentiable from UV experiments due to the presence of multiple competing structures in such long constructs. Due to the significant differences in structure populations while a long construct was either free (in UV, CD, NMR experiments) or constrained within a longer chain of genomic DNA (in G4-seq experiments), we expected the discrepancy between the biophysical and G4-seq experiments to be significant. In any case, while using the same concentration of the dissolved DNA, and the same number of scans, NMR did show more pronounced signals for Hoogsteen-paired bases in sequence **c**, as compared to **b**. Furthermore, the CD melting curves, though with T_m not directly differentiable, still favor sequence **c**, in terms of a thermal stability. The CD melting for sequence **c** has a well pronounced single intersection point with the 0 value of molar ellipticity, reflective of a single structure present.

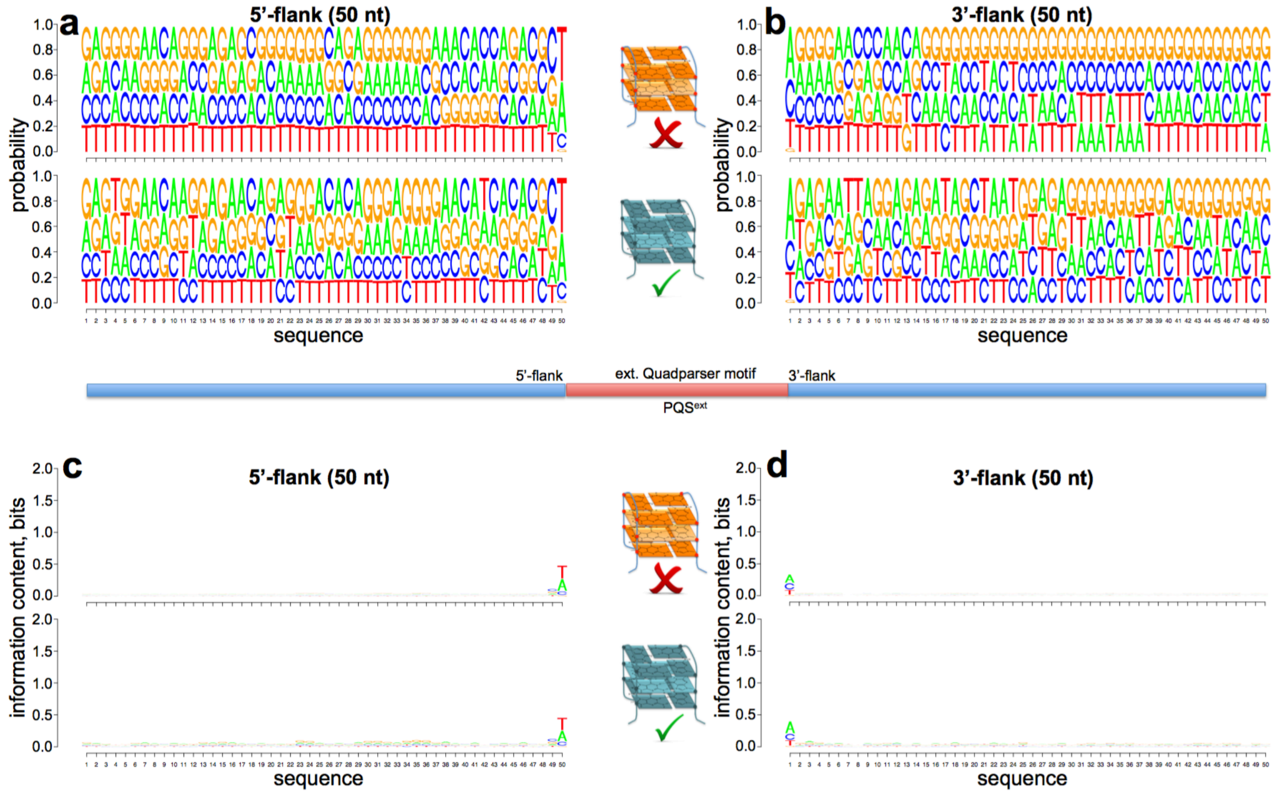

Figure S15 | Sequence logo representations of 50-nt-long 5'- and 3'-flanking regions around sequences of extended putative quadruplex motif. The plots **a** and **b** are sequence logos constructed using base probabilities, whereas **c** and **d** depict the bases in a more meaningful, information content, scale[11]. The schematic representation of the extended PQS motif[5,12,13] (red) with its flanks (blue) is shown in between the two rows of plots, where the plots **a** and **c** are for the 5'-flank and the plots **b** and **d** are for the 3'-flank around PQS. Sequence logos are created for the flanking regions of PQS motifs that either do (> 18 *mm%*, bottom subplots in **a**-**d**) or do not (< 18 *mm%*, top subplots in **a**-**d**) form actual G4 structures as assessed through the G4-seq experiment[6] performed on the human genome. The analysis demonstrates the absence of specific overall sequence motifs or features within the flanks that becomes visible by examining the flanking regions of stable G4 structures, and of PQS motifs that do not form stable G4s (no marked differences between the stable-G4 and weak-G4 plots). Also apparent from the examination of a link between simple sequence features (base contents) and the G4-seq *mm%*, the individual features seem to be very weakly associated with the overall propensity of G4 formation. Therefore, only high-level (hyper-dimensional) methodologies, which combine many weak features to assemble a stronger predictor, could capture the sequence vs. G4-formation link at a sufficiently high level of precision.

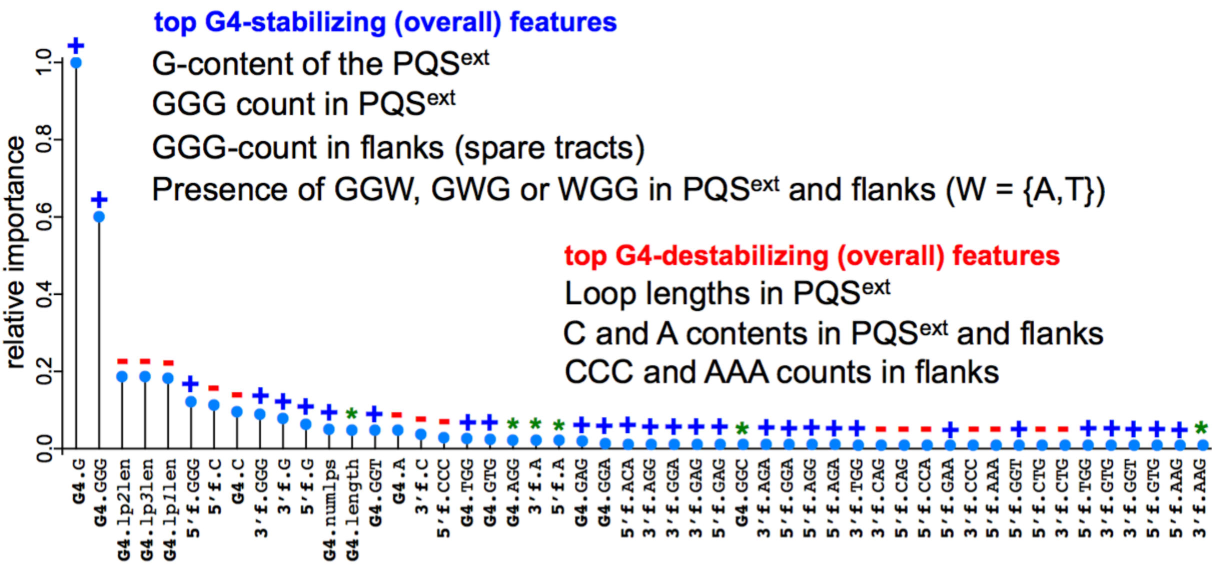

Figure S16 | Top 50 most influential sequence-based features as judged from the final Quadron model. The feature names contain prefixes G4, 5'f and 3'f and denote the features extracted from the PQS, 5'-flank and 3'-flank segments of DNA respectively. The single-letter suffixes denote the singleton contents; three-letter suffixes denote the corresponding triad counts; the suffixes *numlps*, *lp1len*, *lp2len*, *lp3len*, *length*, *lp1efe*, *lp2efe* and *lp3efe* (**Table S3**) denote the number of loops in the extended PQS definition, length of the first, second and third loops, overall length of PQS, ensemble averaged free energies for the sequences of the first, second and third loops. Blue (+), red (-) and green (*) marks highlight the features as generally G4-stabilising, generally G4-destabilising, and more complex respectively. The most pronounced features are summarised on the plot. Please note, that all the used features passed the criterion of not cross-correlating with each other; i. e. the overall G-content in all the PQSs does not correlate too strongly with the GGG count in the same sequences. Furthermore, any single feature presents no significant correlation with the stability of G-quadruplex structure, hence, it is their multiplexed combination through a hyper-dimensional machine learning model that has produced a predictor with high performance.

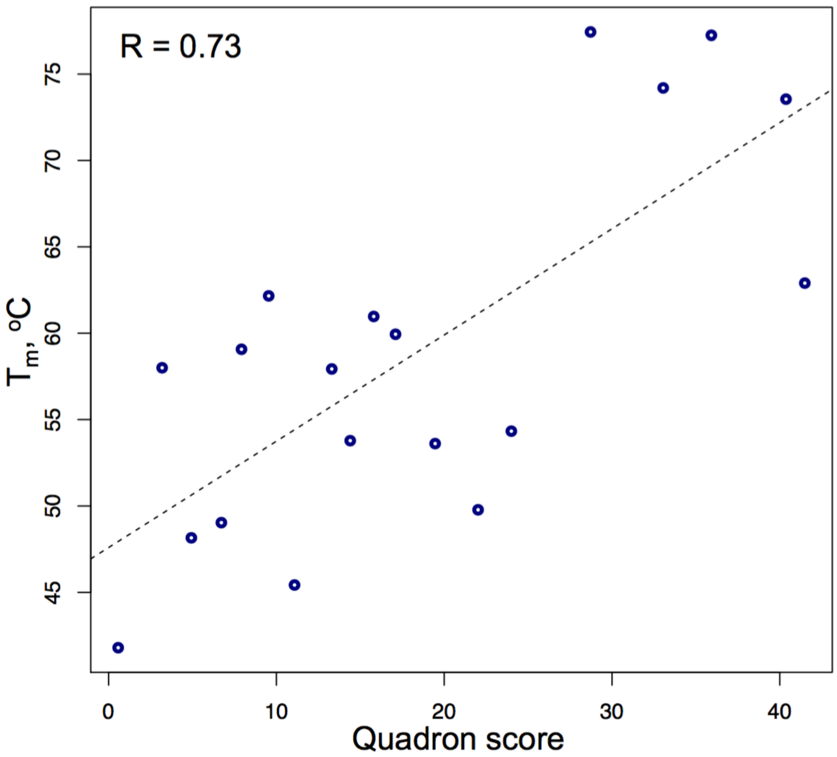

Figure S17 | UV-determined melting temperatures of the randomly picked sequences from *C. elegans* as compared to the computed Quadron scores. The G4 structure melting temperatures (T_m) were obtained from the minimum of the first derivative of the melting curves while following the absorbance at 295 nm[10]. The values reflected in the plot are summarised in **Table S4**, along with the full information on the selected quadruplex sequences and their genomic coordinates in *C. elegans*. Despite the different nature and context of the short-sequence-based UV spectroscopy and genomic G4-seq-based Quadron prediction, a reasonable (Pearson's R = 0.73) correlation can be noted between the T_m and Quadron scores. This demonstrates how, using Quadron, we can obtain novel insight about G4 stability for sequences in genomes, where experimental G4-seq data are not available. The linear trendline is shown on the plot as a dashed line.

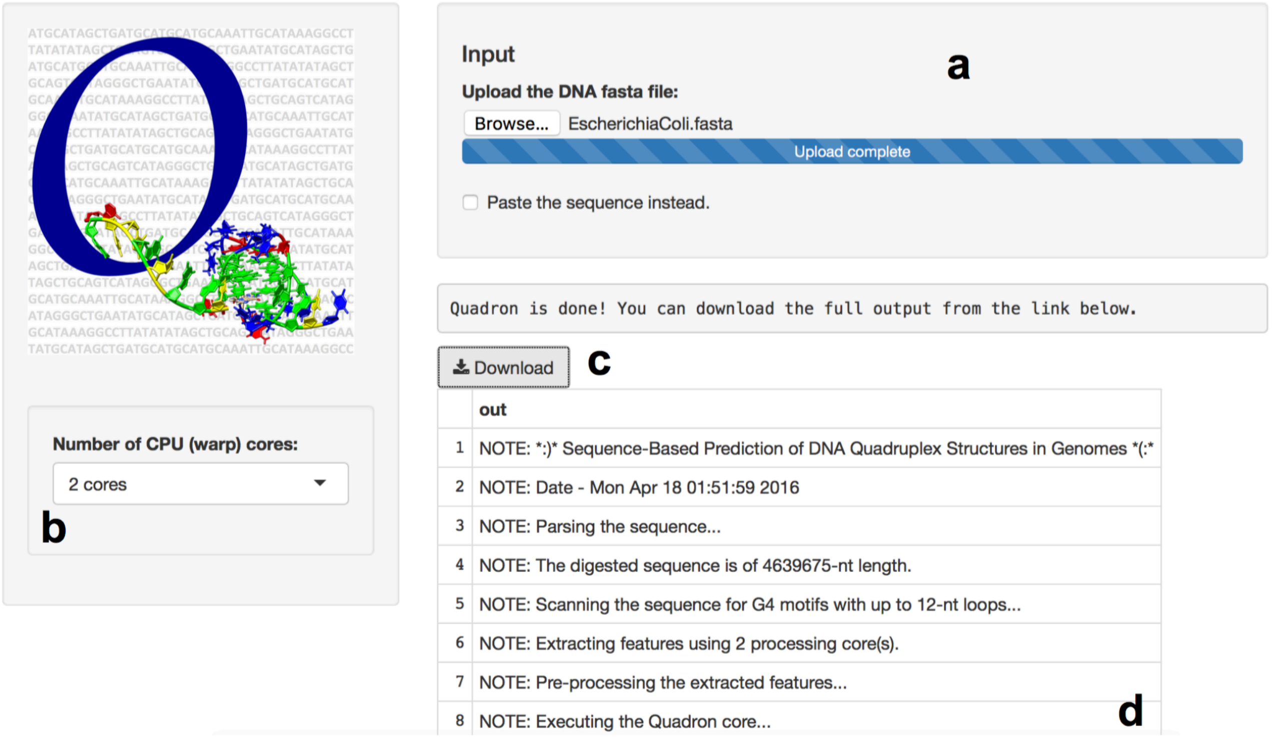

Figure S18 | Graphical user interface (GUI) of the Quadron program. The program accepts either FASTA files with a DNA sequence, or can provide a separate box to paste a sequence directly (useful for small queries) (**a**). The Quadron algorithm is parallelized, hence more than one processing cores (**b**) can be utilized to speed up the computations. The output can then be loaded into any directory (**c**), with a few friendly messages, about the progress of the computation, displayed on fly (**d**).

Table S1 | Sequences representing varying G4-seq *mm%* levels selected for detailed biophysical characterisation. The CD, NMR and UV melting experiments are detailed in **Methods** and **Figures S11-S13**. The experiments have been done on the PQSs only, outside the genomic context captured in the G4-seq experiment[6]. The sequences are brought in conjunction with their G4-seq *mm%* values, genomic location (chr - chromosome, str - strand, pos - genomic coordinate of the sequence border with the smaller value) and ID, as used in **Figures S11** and **S12**. The coloured rows (**b**, **e**, **h**, **i**, **l** and **m**) correspond to the example spectra shown in **Figure 3**. The sequence **c** had too weak UV-melting curve to robustly decipher its melting temperature (hence, n.d.).

ID	G4-seq *mm%*	chr	str	pos	PQS	T_m (°C)
**a**	0.8	10	-	60432001	**GGG**ACT**GGG**AGGA**GGG**AGAAAT**GGG**	50.9
**b**	0.9	5	-	178492127	**GGG**AATAAC**GGG**AAGCT**GGG**TGCA**GGG**	52.7
**c**	0.9	5	-	120045316	**GGG**AA**GGG**TAATAAAGAGTA**GGG**AGGAAGA**GGG**	n.d
**d**	4.3	4	+	164721276	**GGG**AACTACTA**GGG**CT**GGG**AACAA**GGGG**	46.4
**e**	5.2	10	-	100402034	**GGG**ATTAGTGAT**GGG**CAT**GGG**AT**GGG**	50.8
**f**	5.8	7	+	75855277	**GGGG**TCCCCA**GGG**CA**GGG**CT**GGG**	45.1
**g**	9.3	6	-	1973720	**GGG**ATGAA**GGG**TGTGGTTCTCA**GGG**A**GGG**	45.6
**h**	10.1	4	+	7326729	**GGG**A**GGG**CAGAAGGCAGT**GGGG**ATGT**GGG**	53.1
**i**	20.0	22	+	16467917	**GGG**C**GGG**TC**GGGGG**CACCGCGA**GGG**	64.0
**j**	20.0	5	-	120826158	**GGG**AGGA**GGGGG**CCAC**GGGG**AT**GGGG**	78.8
**k**	30.0	1	+	203401919	**GGG**TGGA**GGGGG**A**GGG**AGTT**GGGGGG**	77.4
**l**	30.7	13	-	105003840	**GGGG**CCA**GGG**T**GGGG**T**GGGG**T**GGG**	87.6
**m**	39.1	17	+	46088667	**GGGG**A**GGG**TAGAAAA**GGGG**T**GGGG**	75.9

Table S2 | One of the studied G4 sequence, separately and within the context of two different flanking DNA. The sequences are brought in conjunction with their G4-seq *mm%* values, genomic location (chr - chromosome, str - strand, pos - genomic coordinate of the sequence border with the smaller value) and ID, as used in **Figure S14**.

ID	G4-seq *mm%*	chr	str	pos	PQS
seq. **a**	na	na	na	na	**GGG**A**GGG**A**GGG**A**GGG**
seq. **b**	7.4	5	+	49835788	AGAAAGAAGGAAACAAAGAAACAAAGGAAGAGA<br>AGGCAGGAAGGAAGGAA**GGG**A**GGG**A**GGG**A**GGG**A<br>AGGAAGGAAAAGAAAGAAAGAAAGAAAGAAAGA<br>AAGAAAGAAAGAAAGA
seq. **c**	45.7	7	-	25192340	GGTGACAGAGAGACTCTGTCTCAAAAAAAAAAA<br>AAAAGAGAGACAACGAA**GGG**A**GGG**A**GGG**A**GGG**A<br>GGAAAAGGAAGAGAGAGAGAAAGAGGAAGGGAG<br>GGAGGAAGGAAAGAAG

Table S3 | Feature importance and crude directionality in defining G4-structure stabilities. The relative importance values were normalized for the most influential feature to have a value of 100. Hence, for the rest of the features, importance values represent the fractions (%) of the importance from the most influential feature. As a crude estimate for the directionality of each feature, i. e. whether an increase in a given feature value leads to an increase in G4 stability ("+" in **Figure 5D**) or vice versa ("-" in **Figure 5D**), the training dataset was further analyzed to retrieve the mean (MN) and standard deviation (SD) values of each feature in strong-G4 (ON, *mm%* > 18) vs. weak-G4 (OFF, *mm%* < 18) clusters (**Figure S1**). Further, the ON/OFF ratio and ON-OFF difference were calculated for each feature as presented in the table. The *p-values* reflecting the significance of the difference is estimated based on the comparison of ON and OFF distributions *via* a two-sided Mann-Whitney test. For the ON/OFF ratio, values that are significantly greater than 1, hence are positively correlated with G4 stability, are highlighted in blue. Values that are significantly less than 1, hence are negatively correlated with G4 stability, are highlighted in red. Green colour is used for the values that are either close to 1, or the difference is not significant as judged based on the *p-value*. The colouring is used for the top 50 features only (**Figure 5D**). The feature names should be deciphered as described in **Methods**.

Feature	Rel. Imp	ON.MN	ON.SD	OFF.MN	OFF.SD	ON/OFF	ON-OFF	p-value
G4.G	100.0000	0.6508	0.0850	0.5286	0.0723	**1.2311**	0.1222	0.00E+00
G4.GGG	60.2118	8.9854	5.5121	5.6674	2.4321	**1.5855**	3.3180	0.00E+00
G4.lp2len	18.7066	3.6148	3.1884	6.2601	3.2365	**0.5774**	-2.6453	0.00E+00
G4.lp3len	18.6704	3.6388	3.1831	6.3448	3.2643	**0.5735**	-2.7059	0.00E+00
G4.lp1len	18.2796	4.5058	3.4259	6.4533	3.2276	**0.6982**	-1.9475	0.00E+00
5'f.GGG	12.1290	1.5435	1.6401	0.7570	1.0479	**2.0389**	0.7865	0.00E+00
5'f.C	11.2899	0.2158	0.0909	0.2425	0.0958	**0.8901**	-0.0266	0.00E+00
G4.C	9.6042	0.0869	0.0634	0.1390	0.0756	**0.6255**	-0.0520	0.00E+00
3'f.GGG	8.9301	1.4516	1.6265	0.8083	1.0742	**1.7958**	0.6433	0.00E+00
3'f.G	7.8474	0.2940	0.0953	0.2534	0.0806	**1.1604**	0.0406	0.00E+00
5'f.G	6.2914	0.2934	0.0931	0.2513	0.0796	**1.1675**	0.0421	0.00E+00
G4.numlps	5.0018	4.5818	3.1125	3.5317	1.1640	**1.2973**	1.0501	0.00E+00
G4.length	4.7670	36.5947	26.0644	35.1827	10.0337	**1.0401**	1.4121	0.00E+00
G4.GGT	4.7634	1.5414	2.0616	0.9913	0.9667	**1.5549**	0.5501	0.00E+00
G4.A	4.7629	0.1388	0.0733	0.1892	0.0766	**0.7337**	-0.0504	0.00E+00
3'f.C	3.7712	0.2129	0.1052	0.2420	0.0982	**0.8799**	-0.0291	0.00E+00
5'f.CCC	2.9663	0.7469	1.3051	1.1493	1.5733	**0.6499**	-0.4023	0.00E+00
G4.TGG	2.7552	2.1219	2.0474	1.6882	1.1618	**1.2569**	0.4337	0.00E+00
G4.GTG	2.5609	1.4649	1.8712	0.8286	0.9984	**1.7679**	0.6363	0.00E+00
G4.AGG	2.2819	2.3476	2.7892	2.1060	1.5854	*1.1147*	0.2416	**2.23E-01**
3'f.A	2.2785	0.2670	0.1026	0.2609	0.0931	**1.0232**	0.0061	1.34E-67
5'f.A	2.2528	0.2854	0.1113	0.2720	0.0976	**1.0492**	0.0134	2.75E-190
G4.GAG	1.9673	1.8133	2.3915	1.3728	1.6606	**1.3209**	0.4405	0.00E+00
G4.GGA	1.4353	2.2450	2.5368	1.8767	1.5864	**1.1962**	0.3683	4.17E-177
5'f.ACA	1.2207	1.6308	2.2894	1.0247	1.3113	**1.5915**	0.6061	6.56E-241
3'f.AGG	1.2124	1.6039	1.5133	1.3118	1.2489	**1.2227**	0.2921	0.00E+00
3'f.GGA	1.1781	1.3696	1.4653	1.0422	1.1114	**1.3141**	0.3274	0.00E+00
3'f.GAG	1.1714	1.5243	1.6061	1.2036	1.3046	**1.2664**	0.3207	0.00E+00

5'f.GAG	1.1703	1.5906	1.5614	1.2316	1.2971	**1.2915**	0.3590	0.00E+00
G4.GGC	1.1048	1.2754	1.7876	1.2755	1.1471	**0.9999**	-0.0001	7.15E-209
3'f.AGA	1.0890	1.3611	1.6064	1.2554	1.3837	**1.0841**	0.1056	2.32E-43
5'f.GGA	1.0843	1.7000	1.5169	1.1137	1.1353	**1.5264**	0.5863	0.00E+00
5'f.AGG	1.0777	1.6480	1.4459	1.2766	1.1983	**1.2909**	0.3714	0.00E+00
5'f.AGA	1.0618	1.4404	1.6250	1.3253	1.4029	**1.0868**	0.1150	5.44E-45
3'f.TGG	1.0455	1.3371	1.2302	1.1800	1.0674	**1.1331**	0.1571	3.09E-181
3'f.CAG	1.0307	1.3373	1.1429	1.4348	1.2025	**0.9321**	-0.0975	9.06E-89
5'f.CAG	1.0161	1.3273	1.1236	1.4340	1.2037	**0.9256**	-0.1067	8.33E-103
5'f.CCA	1.0000	0.8222	0.9563	1.1565	1.1024	**0.7109**	-0.3343	0.00E+00
5'f.GAA	0.9977	1.5507	1.6718	1.0697	1.1734	**1.4497**	0.4810	0.00E+00
3'f.CCC	0.9653	0.8859	1.5364	1.0862	1.5338	**0.8156**	-0.2003	0.00E+00
5'f.AAA	0.9493	1.1405	2.1508	1.2056	1.7817	**0.9460**	-0.0651	7.82E-260
5'f.GGT	0.9273	0.7699	0.9561	0.6733	0.8242	**1.1436**	0.0967	5.53E-75
5'f.CTG	0.9163	1.0990	1.1470	1.2649	1.1656	**0.8688**	-0.1660	0.00E+00
3'f.CTG	0.9104	1.1222	1.1804	1.3641	1.1928	**0.8227**	-0.2419	0.00E+00
5'f.TGG	0.9103	1.3288	1.1942	1.1157	1.0330	**1.1910**	0.2131	0.00E+00
3'f.GTG	0.8894	1.0917	1.2580	0.9298	1.0845	**1.1741**	0.1619	6.96E-275
3'f.GGT	0.8882	0.9107	0.9514	0.7145	0.8445	**1.2746**	0.1962	0.00E+00
5'f.GTG	0.8795	1.0041	1.3703	0.8991	1.0817	**1.1167**	0.1050	8.11E-21
5'f.AAG	0.8676	1.1461	1.4121	1.0619	1.1374	**1.0794**	0.0843	8.41E-03
3'f.AAG	0.8557	1.0062	1.3826	1.0331	1.1542	**0.9740**	-0.0268	2.26E-146
5'f.GCC	0.8496	0.7817	1.0448	0.9139	1.0452	0.8554	-0.1321	0.00E+00
3'f.AAA	0.8117	1.0845	1.6231	1.1134	1.6519	0.9740	-0.0289	2.69E-01
3'f.GCA	0.8045	1.0170	1.0006	0.8946	0.9682	1.1369	0.1224	4.80E-257
3'f.GAA	0.8031	0.9799	1.3420	0.9595	1.1099	1.0213	0.0204	8.67E-36
3'f.GGC	0.7944	0.9603	1.1257	0.9003	1.0114	1.0666	0.0600	2.07E-07
5'f.GGC	0.7737	0.9487	1.1218	0.9082	1.0139	1.0445	0.0404	9.30E-01
5'f.CCT	0.7736	0.7799	1.0021	1.1002	1.1552	0.7089	-0.3203	0.00E+00
G4.lp1efe	0.7686	-0.0313	0.1363	-0.0545	0.1829	0.5739	0.0232	0.00E+00
3'f.AGC	0.7540	0.9763	0.9495	0.9398	0.9894	1.0388	0.0365	5.93E-57
5'f.CAC	0.7426	1.1656	1.5592	0.8872	1.1040	1.3137	0.2783	3.10E-76
3'f.CCT	0.7358	0.9689	1.0347	1.1390	1.1516	0.8506	-0.1701	6.02E-287
5'f.AGC	0.7348	0.8215	0.9581	0.9635	0.9942	0.8527	-0.1419	0.00E+00
3'f.CCA	0.7318	0.8050	0.9719	1.0683	1.0733	0.7535	-0.2633	0.00E+00
3'f.GCC	0.7126	0.8096	1.1005	0.8658	1.0071	0.9351	-0.0562	1.78E-180
3'f.TGA	0.6996	0.9639	0.9384	0.9766	0.9684	0.9870	-0.0127	6.74E-01
5'f.GCA	0.6962	0.8320	0.9734	0.9380	0.9889	0.8870	-0.1060	1.37E-225
G4.CTG	0.6961	0.6920	1.2792	0.8749	0.9578	0.7909	-0.1829	0.00E+00
5'f.TCT	0.6921	0.6798	0.9568	0.8740	1.0541	0.7778	-0.1942	0.00E+00
5'f.TGT	0.6888	0.7482	1.2530	0.8485	1.0798	0.8818	-0.1003	0.00E+00
5'f.TGA	0.6755	1.0266	0.9649	0.9553	0.9551	1.0746	0.0712	2.93E-105
3'f.TTT	0.6755	0.7498	1.5664	0.9403	1.5685	0.7973	-0.1906	0.00E+00
3'f.ACA	0.6725	0.7067	0.9454	0.8462	1.0112	0.8351	-0.1395	0.00E+00
3'f.TGC	0.6719	0.8778	0.9170	0.8911	0.9607	0.9850	-0.0134	3.48E-01
5'f.CTC	0.6651	0.6947	0.9692	0.9275	1.1040	0.7490	-0.2328	0.00E+00
3'f.GCT	0.6635	0.8319	0.9389	0.9018	0.9500	0.9225	-0.0699	1.54E-102
5'f.GCT	0.6629	0.7811	0.9574	0.8911	0.9577	0.8766	-0.1100	6.02E-294

3'f.ATG	0.6509	1.0819	1.2362	0.8504	0.9465	1.2723	0.2316	2.11E-293
3'f.TCT	0.6487	0.6792	0.9857	0.9418	1.1076	0.7212	-0.2626	0.00E+00
5'f.TCC	0.6287	0.6146	0.8725	0.8916	1.0167	0.6893	-0.2770	0.00E+00
G4.CGG	0.6267	0.5924	1.5278	0.3494	0.7253	1.6955	0.2430	0.00E+00
5'f.TGC	0.6182	0.6787	0.8954	0.8422	0.9498	0.8058	-0.1635	0.00E+00
G4.AAG	0.6134	0.5595	1.2228	0.7514	0.9806	0.7446	-0.1919	0.00E+00
G4.CAG	0.6106	0.6154	1.1456	0.9363	0.9883	0.6572	-0.3210	0.00E+00
5'f.ATG	0.6067	0.9173	1.0321	0.8398	0.9363	1.0924	0.0776	5.57E-68
3'f.TGT	0.6009	0.7919	1.1118	0.8575	1.0590	0.9236	-0.0655	1.05E-94
5'f.TTT	0.5907	0.6612	1.3567	0.8040	1.3673	0.8224	-0.1428	0.00E+00
G4.lp3efe	0.5895	-0.0214	0.1266	-0.0528	0.1873	0.4059	0.0314	0.00E+00
5'f.TCA	0.5796	0.7332	0.8080	0.8262	0.8845	0.8875	-0.0930	9.95E-127
G4.AGA	0.5757	0.4308	1.0258	0.5931	0.9693	0.7263	-0.1623	0.00E+00
G4.lp2.efe	0.5681	-0.0217	0.1267	-0.0489	0.1769	0.4429	0.0273	0.00E+00
3'f.AGT	0.5615	0.7190	0.8276	0.7687	0.8711	0.9353	-0.0498	2.70E-35
5'f.ACC	0.5590	0.4988	0.7092	0.6427	0.8171	0.7761	-0.1439	0.00E+00
G4.GCG	0.5439	0.4442	1.2248	0.2002	0.6183	2.2191	0.2440	0.00E+00
G4.GAA	0.5397	0.5572	1.2434	0.7526	0.9846	0.7404	-0.1953	0.00E+00
G4.GCC	0.5381	0.3828	0.7682	0.5273	0.7568	0.7259	-0.1445	0.00E+00
5'f.CTT	0.5316	0.5952	0.8256	0.7614	0.9071	0.7817	-0.1662	0.00E+00
3'f.TAG	0.5022	0.7018	0.9064	0.5322	0.7559	1.3187	0.1696	0.00E+00
5'f.CAA	0.4975	0.7143	0.8159	0.7869	0.8863	0.9077	-0.0726	3.67E-67
G4.GCA	0.4787	0.4904	0.9725	0.6546	0.8235	0.7491	-0.1643	0.00E+00
3'f.GAT	0.4350	0.7449	0.9723	0.6158	0.8024	1.2097	0.1291	4.02E-214
5'f.AAT	0.4223	0.7356	0.9267	0.7386	0.9722	0.9959	-0.0030	1.88E-05
3'f.CCG	0.3977	0.3219	0.7649	0.2302	0.5538	1.3982	0.0917	6.25E-99
G4.CCC	0.3590	0.1260	0.5133	0.3224	0.7172	0.3908	-0.1964	0.00E+00
3'f.CGC	0.3528	0.2841	0.7390	0.1916	0.5488	1.4833	0.0926	2.24E-191
5'f.CCG	0.3490	0.2879	0.6782	0.2317	0.5589	1.2422	0.0561	3.59E-49
G4.GCT	0.3425	0.4531	1.0282	0.5877	0.7952	0.7709	-0.1346	0.00E+00
3'f.ATA	0.3190	0.6993	1.0478	0.5210	0.8863	1.3423	0.1783	0.00E+00
3'f.GCG	0.3060	0.3382	0.8103	0.2028	0.5754	1.6672	0.1353	0.00E+00
G4.TTG	0.2828	0.4156	0.7296	0.4125	0.6473	1.0076	0.0031	2.09E-02
5'f.CGC	0.2796	0.2507	0.6757	0.1943	0.5634	1.2902	0.0564	1.76E-80
5'f.GCG	0.2757	0.3220	0.7972	0.1992	0.5726	1.6166	0.1228	0.00E+00
5'f.CGG	0.2613	0.3515	0.7511	0.2312	0.5633	1.5200	0.1202	0.00E+00
3'f.TAT	0.2079	0.4262	0.7595	0.4682	0.8280	0.9103	-0.0420	5.30E-17
G4.GTT	0.2045	0.3537	1.2941	0.3033	0.5658	1.1663	0.0504	1.31E-56
G4.TGT	0.1996	0.4016	0.9696	0.3050	0.6077	1.3167	0.0966	2.12E-151
G4.AGC	0.1911	0.2413	0.7321	0.3600	0.6076	0.6702	-0.1187	0.00E+00
3'f.TAC	0.1908	0.4195	0.6512	0.4022	0.6722	1.0431	0.0173	1.14E-41
G4.TGC	0.0875	0.1782	0.5642	0.2640	0.5319	0.6749	-0.0858	0.00E+00
G4.ATA	0.0000	0.0870	0.3624	0.1479	0.4354	0.5879	-0.0610	0.00E+00

Table S4 | Sequences randomly selected from *C. elegans* spanning varying stability scores, determined computationally *via* Quadron. The sequences are brought in conjunction with their Quadron scores, genomic location (chr - chromosome, str - strand, pos - genomic coordinate of the sequence border with the smaller value), ID and the UV-determined melting temperatures (T_m). Please note, that the UV-melting experiments have been done on the PQS sequences only, outside the genomic context captured in the Quadron calculations.

ID	Quadron score	chr	str	pos	PQS	T_m (°C)
**A**	0.57	X	+	6168404	**GGG**AATGCTT**GGG**AATT**GGG**AATTAAATT**GGG**	41.8
**B**	3.19	IV	-	5269541	**GGG**AGACA**GGG**ATGCA**GGGGG**ATCATTGT**GGG**	58.0
**C**	4.93	IV	+	7559173	**GGGG**ACCGT**GGGG**ATTGGA**GGG**ACT**GGG**	48.2
**D**	6.72	I	+	3376650	**GGG**ATTTCTAA**GGG**TT**GGGG**AGATC**GGG**	49.0
**E**	7.92	IV	+	13008156	**GGG**TTT**GGG**CTT**GGG**TTTAGGCTT**GGG**	59.1
**F**	9.54	X	+	13250248	**GGG**CGAGA**GGG**AGC**GGG**AGAAGC**GGG**T**GGG**	62.2
**G**	11.08	X	-	14862041	**GGG**CTCATTACAC**GGG**AC**GGG**AAAA**GGGGGG**	45.4
**H**	13.3	I	-	11790162	**GGG**TTAGGA**GGG**AATTAA**GGGGG**CT**GGGG**	57.9
**I**	14.4	V	+	1693585	**GGG**ATTT**GGG**AA**GGG**ATT**GGG**	53.8
**J**	15.8	X	+	9771776	**GGG**AC**GGG**AAGACGCGGT**GGG**AT**GGG**	61.0
**K**	17.1	IV	-	12826470	**GGG**ATT**GGG**AAAC**GGGG**AGAAGTT**GGGGG**	59.9
**L**	19.46	I	+	7711640	**GGGG**TT**GGG**AGTGAGTGA**GGG**AAGT**GGG**	53.6
**M**	22.02	I	+	14183102	**GGG**AGA**GGG**ATACTGTA**GGG**A**GGG**	49.8
**N**	24	V	-	18415806	**GGG**TACTTGGTCT**GGG**CCAA**GGGGG**CTT**GGG**	54.3
**O**	28.72	IV	+	1811299	**GGGG**AGGCAAGA**GGGGG**C**GGG**C**GGG**	77.4
**P**	33.05	III	-	2980298	**GGG**A**GGG**CAT**GGG**A**GGGGGG**	74.2
**Q**	35.92	IV	+	14339135	**GGG**CTT**GGG**T**GGG**AT**GGGG**	77.2
**R**	40.37	X	-	8360047	**GGG**T**GGG**CCATAATCAT**GGG**T**GGGG**	73.6
**S**	41.49	X	+	16398498	**GGG**T**GGGG**T**GGG**TTTGTGTGTATT**GGG**	62.9

SUPPORTING REFERENCES

1. Friedman, J. H. Greedy function approximation: a gradient boosting machine. *IMS Reitz Lecture* 1–39 (1999), accessible from http://statweb.stanford.edu/~jhf/ftp/trebst.pdf.
2. Kuhn, M. & Johnson, K. *Applied predictive modeling*. (Springer, New York, 2013).
3. Natekin, A. & Knoll, A. Gradient boosting machines, a tutorial. *Front. Neurorobot.* **7,** 21 (2013).
4. R Core Team. R: a language and environment for statistical computing. *R Foundation for Statistical Computing, Vienna, Austria* (2015).
5. Maizels, N. & Gray, L. T. The G4 genome. *PLoS Genet.* **9,** e1003468 (2013).
6. Chambers, V. S. *et al.* High-throughput sequencing of DNA G-quadruplex structures in the human genome. *Nat. Biotech.* **33,** 877–881 (2015).
7. Masiero, S. *et al.* A non-empirical chromophoric interpretation of CD spectra of DNA G-quadruplex structures. *Org. Biomol. Chem.* **8,** 2683–2692 (2010).
8. Adrian, M., Heddi, B. & Phan, A. T. NMR spectroscopy of G-quadruplexes. *Methods* **57,** 11–24 (2012).
9. Sengar, A., Heddi, B. & Phan, A. T. Formation of G-quadruplexes in poly-G sequences: structure of a propeller-type parallel-stranded G-quadruplex formed by a G_{15} stretch. *Biochem.* **53,** 7718–7723 (2014).
10. Mergny, J. L., Phan, A. T. & Lacroix, L. Following G-quartet formation by UV-spectroscopy. *FEBS Lett.* **435,** 74–78 (1998).
11. Schneider, T. D. & Stephens, R. M. Sequence logos: a new way to display consensus sequences. *Nucl. Acids Res.* **18,** 6097–6100 (1990).
12. Huppert, J. & Balasubramanian, S. Prevalence of quadruplexes in the human genome. *Nucl. Acids Res.* **33,** 2908–2916 (2005).
13. Todd, A. K., Johnston, M. & Neidle, S. Highly prevalent putative quadruplex sequence motifs in human DNA. *Nucl. Acids Res.* **33,** 2901–2907 (2005).
